# Antibacterial Secondary Metabolites from Marine-Derived Fungus *Aspergillus* sp. IMCASMF180035

**DOI:** 10.3390/antibiotics10040377

**Published:** 2021-04-03

**Authors:** Fuhang Song, Rui Lin, Na Yang, Jia Jia, Shangzhu Wei, Jiahui Han, Jiangpeng Li, Hongkai Bi, Xiuli Xu

**Affiliations:** 1 School of Light Industry, Beijing Technology and Business University, Beijing 100048, China; 2 School of Ocean Sciences, China University of Geosciences, Beijing 100083, China; linrui520@126.com (R.L.); wert0715@163.com (S.W.); 15632779760@163.com (J.H.); culjp2016@163.com (J.L.); 3 CAS Key Laboratory of Experimental Marine Biology, Institute of Oceanology, Chinese Academy of Sciences, Qingdao 266071, China; yangna@qdio.ac.cn; 4 Laboratory for Marine Biology and Biotechnology, Qingdao National Laboratory for Marine Science and Technology, Qingdao 266071, China; 5 Department of Pathogen Biology, Jiangsu Key Laboratory of Pathogen Biology, Nanjing Medical University, Nanjing 211166, China; jiajia@njmu.edu.cn (J.J.); hkbi@njmu.edu.cn (H.B.)

**Keywords:** marine-derived fungus, *Aspergillus* sp., natural products, anti-*Staphylococcus aureus*, anti-*Helicobacter pylori*

## Abstract

Four new secondary metabolites, including one *spiro*[anthracenone-xanthene] derivative aspergiloxathene A (**1**), one penicillide analogue, Δ^2′^-1′-dehydropenicillide (**2**), and two new phthalide derivatives, 5-methyl-3-methoxyepicoccone (**3**) and 7-carboxy-4-hydroxy-6-methoxy-5-methylphthalide (**4**), together with four known compounds, yicathin C (**5**), dehydropenicillide (**6**), 3-methoxyepicoccone (**7**), 4-hydroxy-6-methoxy-5-methylphthalide (**8**), were identified from the marine-derived fungus *Aspergillus* sp. IMCASMF180035. Their structures were determined by spectroscopic data, including high-resolution electrospray ionization mass spectrometry (HRESIMS), 1D and 2D nuclear magnetic resonance (NMR) techniques. Compound **1** was identified as the first jointed molecule by xanthene and anthracenone moieties possessing an unprecedented carbon skeleton with spiro-ring system. All compounds were evaluated activities against *Staphylococcus aureus*, methicillin resistant *S. aureus* (MRSA), *Escherichia coli*, *Escherichia faecium*, *Pseudomonas aeruginosa*, and *Helicobacter pylori.* Compound **1** showed significant inhibitory effects against *S. aureus* and MRSA, with minimum inhibitory concentration (MIC) values of 5.60 and 22.40 µM. Compounds **2** and **6** exhibited potent antibacterial activities against *H. pylori*, with MIC values of 21.73 and 21.61 µM, respectively.

## 1. Introduction

Overuse of antibiotics has led to the emergence and maintenance of drug resistance. The ESKAPE (*Enterococcus faecium*, *Staphylococcus aureus*, *Klebsiella pneumoniae*, *Acinetobacter baumanii*, *Pseudomonas aeruginosa*, and *Enterobacter species*) pathogens are responsible for a variety of infectious diseases with a wide range of drug resistance to current clinical drugs [1]; therefore, there is urgent need to develop novel antibiotics [2,3]. Natural products have historically played an important role in the development of novel antibacterial agents [4]. Natural products characterized from marine-derived organisms are considered more and more important for drug development [5,6,7]. In recent years, more than 1000 new compounds were identified from organisms living in marine habitats per annum [8,9,10], which suggests the great potential of discovering new chemical entries from marine-derived organisms.

**Figure 1:**
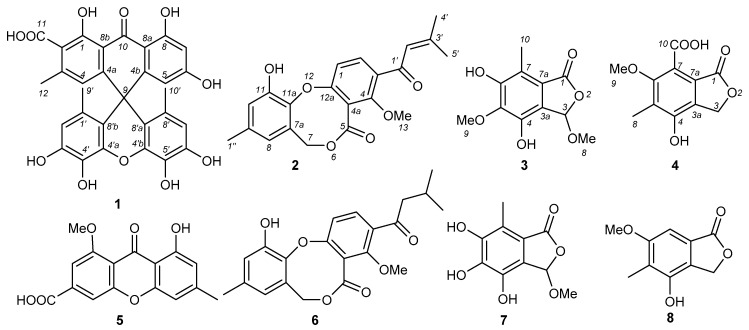
Chemical structures of **1**–**8**.

Fungi of the *Aspergillus* genus isolated from a marine environment have been proven to be rich sources for discovering new chemical entries with antibacterial and antifungal [11,12,13,14,15,16,17,18], inflammatory [19], antiviral [20,21], and antitumor [22,23] activities. During our ongoing search for novel bioactive secondary metabolites against bacteria and mycobacteria from marine-derived fungi, the crude extract of the fungus *Aspergillus* sp. IMCASMF180035, isolated from a sediment sample collected from the intertidal zones of the Yellow Sea in Qingdao, China, showed antibacterial activity against *S. aureus* with a minimum inhibitory concentration (MIC) value of 25 µg/mL. Further chemical investigation on the ethyl acetate extract led to the identification of eight natural products (Figure 1), including four new secondary metabolites, aspergiloxathene A (**1**), Δ^2′^-1′-dehydropenicillide (**2**), 5-methyl-3-methoxyepicocc- one (**3**), and 7-carboxy-4-hydroxy-6-methoxy-5-methylphthalide (**4**), together with four known compounds, yicathin C (**5**), 1′-dehydropenicillide (**6**), 3-methoxyepicoccone (**7**), and 4-hydroxy-6-methoxy-5-methylphthalide (**8**). To the best of our knowledge, aspergiloxathene A (**1**) is the first natural product with xanthene and anthracenone fragments possessing an unprecedented carbon skeleton with a spiro-ring system. Herein, we report the isolation, structural elucidation, and biological activities of these compounds.

## 2. Results and Discussion

### 2.1. Culture of Fungus and Isolation of Compounds

*Aspergillus* sp. IMCASMF180035 was inoculated into 1 L flasks with rice solid medium and incubated stationary at 28 °C for 30 days. The cultures and medium were extracted by EtOAc:MeOH and followed by a series of purification methods, such as partition, reduced normal phase silica chromatography, Sephadex LH-20 chromatography, and high-performance liquid chromatography according to our previous report [11,12] to yield compounds **1**–**8**.

### 2.2. Structure Elucidation

**Figure 2:**
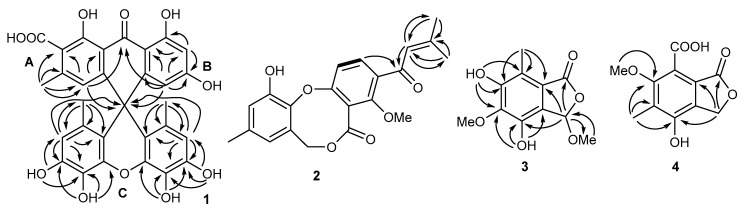
¹H-¹H correlation spectroscopy (¹H-¹H COSY) and key heteronuclear multiple bond correlation (HMBC) correlations of **1**–**4**.

Compound **1** was isolated as a light yellow powder. The molecular formula of **1** was determined to be C_30_H_22_O_11_ based on the high-resolution electrospray ionization mass spectrometry (HRESIMS) spectrum (*m/z* [M + H]^+^ 559.1235, calcd for C_30_H_22_O_11_^+^, 559.1235), accounting for 20 degrees of unsaturation (Supplementary Figure S1). The ^1^H NMR and ¹H-¹H correlation spectroscopy data of **1** (Table 1 and Supplementary Figures S2 and S5) demonstrated four aromatic signals at *δ*_H_ 6.28 (1H, s, H-4), 5.90 (1H, d, *J* = 2.0 Hz, H-5), 6.19 (1H, d, *J* = 2.0 Hz, H-7), and 6.15 (2H, s, H-2’, and H-7’); three methyl groups at *δ*_H_ 2.10 (3H, s, H-12) and 1.34 (6H, s, H-9’, and H-10’); and four phenolic hydroxyl groups at *δ*_H_ 9.90 (2H, s, 4’-OH, and 5’-OH) and 8.95 (2H, s, 3’-OH, and 6’-OH). The ^13^C and heteronuclear single quantum coherence (HSQC) spectrum of **1** (Supplementary Figures S3 and S4) indicated 30 carbons signals (Table 1) for one carbonyl at *δ*_C_ 190.2 (C-10); one carboxyl at *δ*_C_ 167.5 (C-11); five sp^2^ methine carbons at *δ*_C_ 123.5 (C-4), 111.7 (C-5), 115.5 (C-2’ and C-7’), and 101.3 (C-7); nineteen sp^2^ quaternary carbons at *δ*_C_ 165.6 (C-6), 163.3 (C-8), 156.6 (C-1), 153.5 (C-4b), 150.6 (C-4a), 144.1 (C-3’ and C-6’), 143.2 (C-3), 137.3 (C-4’a and C-4’b), 131.0 (C-4’ and C-5’), 125.9 (C-1’ and C-8’), 122.6 (C-2), 114.1 (C-8b), 117.7 (C-8’a and C-8’b), and 109.7 (C-8a); one sp^3^ quaternary carbon at *δ*_C_ 45.6 (C-9); and three methyl carbons at *δ*_C_ 19.9 (C-9’ and C-10’) and 19.8 (C-12). All these NMR data suggest that compound **1** contained a multihydroxyl-substituted aromatic compound. The heteronuclear multiple bond correlation (HMBC) correlations (Figure 2 and Supplementary Figures S6–S8) from H-4 to C-2, C-8b, and C-12 and from H-12 to C-2, C-3, and C-4 revealed the moiety of ring A. The HMBC correlations from H-5 to C-4b, C-6, C-7, and C-8a and from H-7 to C-5, C-6, C-8, and C-8a indicated the substructure of ring B. The HMBC correlations from H-4 and H-5 to C-9 and the long-range HMBC correlations from H-4 and H-5 to C-10 suggested that ring A and ring B are connected through C-10 and C-9, as shown in Figure 2. From the integration of H-2’/H-7’, H-3’/H-6’-OH, H-4’/H-5’-OH, and H-9’ and H-10’, moiety C was deduced as a symmetric substructure. The key HMBC correlations from H-2’/H-7’ to C-3’/C-6’, C-4’/C-5’, C-8’b/C-8’a, and C-9’/C-10’ and from H-9’/H-10’ to C-1’/C-8’, C-2’/C-7’, and C-8’b/C-8’a indicated the presence of moiety C. The long-range HMBC correlations from H-2’/H-7’ to C-9 suggested the connections of C-9 to C-8’a and C-8’b. The HMBC crossing peaks from H-3’-OH/H-6’-OH to C-2’/C-7’, C-3’/C-6’, and C-4’/C-5’ and from H-4’-OH/H-5’-OH to C-3’/C-6’, C-4’/C-5’, and C-4a’/C-4b’ revealed the positions of the hydroxyl groups of substructure C. The two rings of the xanthene fragment showed symmetry through a plane of the anthracenone fragment by running Minimize Energy in ChemBio 3D, which was consisted with the zero of optical rotation for **1**. As a result, the structure of compound **1** was assigned as shown in Figure 1 and named as aspergiloxathene A.

Compound **2** was isolated as a light yellow powder. The molecular formula of **1** was determined to be C_21_H_20_O_6_ based on the HRESIMS spectrum (*m/z* [M + H]^+^ 369.1330, calcd for C_21_H_21_O_6_^+^, 369.1333), accounting for twelve degrees of unsaturation (Supplementary Figure S10). The ^1^H NMR data of **2** (Table 1 and Supplementary Figure S11) demonstrated two *ortho* aromatic signals at *δ*_H_ 6.94 (1H, d, *J* = 8.5 Hz, H-1) and 7.72 (1H, d, *J* = 8.5 Hz, H-2), two singlet aromatic protons at *δ*_H_ 6.40 (1H, s, H-8) and 6.87 (1H, s, H-10), and one olefinic proton at *δ*_H_ 6.66 (1H, s, H-2’). In addition, one oxygenated methylene at *δ*_H_ 5.11 (2H, brs, H-7); one methoxyl group at *δ*_H_ 3.91 (3H, s, H-13); and three methyl groups at *δ*_H_ 2.26 (3H, s, H-4’), 2.01 (3H, s, H-5’), and 2.25 (3H, s, H-1’’) were observed in the proton spectrum. The ^13^C and HSQC spectrum of **2** (Supplementary Figures S12 and S13) showed 21 carbons signals (Table 1), including one carbonyl at *δ*_C_ 190.7 (C-1’); one carboxyl at *δ*_C_ 166.4 (C-5); five sp^2^ methine carbons at *δ*_C_ 118.0 (C-1), 134.8 (C-2), 121.2 (C-8), 117.8 (C-10), and 124.1 (C-2’); nine sp^2^ quaternary carbons at *δ*_C_ 134.0 (C-3), 156.9 (C-4), 121.6 (C-4a), 125.9 (C-7a), 135.6 (C-9), 147.4 (C-11), 141.0 (C-11a), 154.0 (C-12a), and 158.8 (C-3’); one oxygenated methylene carbon at *δ*_C_ 69.1 (C-7); one methoxyl carbon at *δ*_C_ 64.3 (C-13); and three methyl carbons at *δ*_C_ 21.7 (C-4’), 28.3 (C-5’), and 21.0 (C-1’’). A detailed analysis of the 1D and 2D NMR data (Figure 2 and Supplementary Figures S11–S15) indicated that **2** is an analogue of penicillide [24]. In the HMBC spectra, the correlations from H-4’ and H-5’ to C-2’ (*δ*_C_ 124.1) and C-3’ (*δ*_C_ 158.8) and from H-2’ to C-4’ and C-5’ revealed the double bond of C-2’ and C-3’. Additionally, the HMBC correlations from H-2 and H-2’ to C-1’ (*δ*_C_ 190.7) indicated the presence of carbonyl at C-1’. Thus, the structure of compound **2** was designed as in Figure 1 and named Δ^2′^-1′-dehydropenicillide.

Compound **3** was isolated as a colorless powder. The molecular formula of **3** was determined to be C_11_H_12_O_6_ based on the HRESIMS spectrum (*m/z* [M + H]^+^ 241.0705, calcd for C_11_H_13_O_6_^+^, 241.0707), accounting for six degrees of unsaturation (Supplementary Figure S17). The ^1^H NMR data of **3** (Table 2 and Supplementary Figure S18) displayed one methine proton at *δ*_H_ 6.31 (1H, s, H-3), one methyl group at *δ*_H_ 2.31 (3H, H-10), and two methoxyl groups at *δ*_H_ 3.40 (3H, s, H-8) and 3.74 (1H, s, H-9) as well as two protons for hydroxyl groups at *δ*_H_ 9.71 (1H, s, 4-OH) and 9.33 (1H, s, 6-OH). The ^13^C and HSQC spectrum of **1** (Supplementary Figures S19 and S20) showed 11 carbon signals (Table 2), including one carboxyl at *δ*_C_ 168.7 (C-1); one acetal signal at *δ*_C_ 100.5 (C-3); six aromatic quaternary carbons at 122.5 (C-3a), 144.0 (C-4), 141.0 (C-5), 151.1 (C-6), 116.1 (C-7), and 119.0 (C-7a); one methyl group at *δ*_C_ 9.5 (C-10); and two methoxyl groups at *δ*_C_ 55.2 (C-8) and 60.3 (C-9). The NMR data revealed the phthalide analogue for compound **3**. The key HMBC correlations (Figure 2 and Supplementary Figure S21) from H-3 to C-8 and H-8 to C-3 revealed the methoxyl group at C-3. Additionally, the HMBC correlations from H-3 to C-1, C-4, and C-7a demonstrated the lactone from C-7a, through C-1/O-2/C-3, to C-3a. The hydroxyl groups at C-4 and C-6 were identified by HMBC correlations from H-4-OH to C-3a, C-4, and C-5 and from H-6-OH to C-5, C-6, and C-7. The methyl group at C-10 was confirmed by HMBC correlations from H-10 to C-6, C-7, and C-7a. The HMBC cross peak from H-9 to C-5 revealed the methoxyl group at C-9 attached to C-5. From the optical rotation, we used c *0.05* in MeOH, and as the data undulated near zero, we could not establish if it was an enantiomeric or racemic form. Based on the analogue of 3-methoxyepicoccone [25], compound **3** was named 5-methyl-3-methoxyepicoccone.

Compound **4** was isolated as a colorless powder. The molecular formula of **4** was determined to be C_11_H_10_O_6_ based on the HRESIMS spectrum (*m/z* [M + H]^+^ 239.0554, calcd for C_11_H_11_O_6_^+^, 239.0550), accounting for seven degrees of unsaturation (Supplementary Figure S23). The ^1^H NMR data of **4** (Table 2 and Supplementary Figure S24) displayed one oxygenated methylene group at *δ*_H_ 5.26 (2H, s, H-3), one methyl group at *δ*_H_ 2.16 (3H, H-8), and one methoxyl groups at *δ*_H_ 3.73 (3H, s, H-9). The ^13^C and HSQC spectrum of **1** (Supplementary Figures S25 and S26) showed 11 carbons signals (Table 2), including two carboxyl groups at *δ*_C_ 168.8 (C-1) and 166.4 (C-10); one oxygenated methylene group at *δ*_C_ 68.0 (C-3); six quaternary carbons at 119.8 (C-3a), 150.7 (C-4), 124.8 (C-5), 155.9 (C-6), 118.7 (C-7), and 128.8 (C-7a); one methyl group at *δ*_C_ 9.8 (C-8); and one methoxyl group at *δ*_C_ 61.8 (C-9). In combination with the HMBC correlations (Figure 2 and Supplementary Figure S27) from H-3 to C-1, C-4, and C-7a; from H-8 to C-4, C-5, and C-6; and from H-9 to C-6, compound **4** was identified as an analogue of phthalide [25] and named 7-carboxy-4-hydroxy-6-methoxy-5-methylphthalide.

Four known secondary metabolites were isolated from *Aspergillus* sp. IMCASMF180035, with three of them identified as yicathin C (**5**) [26], 3-methoxyepicoccone (**7**) [27], 4-hydroxy-6-methoxy-5-methylphthalide (**8**) [28], by comparing the spectroscopic data with each of the reported data. 1′-dehydroxypenicillide (**6**) was characterized by comparing the spectroscopic data with the reported structure and related analogues [24,29].

### 2.3. Biological Activity

All of the compounds were subjected to antibacterial assays against *S. aureus* ATCC 25923, methicillin-resistant *S. aureus* USA300, *E. coli* ATCC 11775, *E. faecium* ATCC 19434, *P. aeruginosa* PAO1, and *H. pylori* G27. Compound **1** exhibited significant antibacterial activities against *S. aureus* and methicillin-resistant *S. aureus* (MRSA), with MIC values of 5.60 and 22.40 mM, respectively (positive control, vancomycin, MIC = 0.35 mM). The compounds containing xanthone or anthracenone moieties showed antibacterial activities against a panel of pathogens [13,30,31]. Compound **1** was the first natural product containing a specific carbon skeleton with a spiro-ring system of xanthene and anthracenone, which may offer the antibacterial activities against drug-sensitive and -resistant *S. aureus* strains. Compounds **2** and **6** exhibited potent antibacterial activities against *H. pylori*, with MIC values of 21.73 and 21.61 mM, respectively (positive control, metronidazole, MIC = 11.68 mM).

## 3. Materials and Methods

### 3.1. General Experimental Procedures

Optical rotations ([α]_D_) were recorded on an Anton Paar MCP 200 Modular Circular Polarimeter (Austria) in a 100 × 2 mm cell at 22 °C. One-dimensional and two-dimensional NMR spectra were measured at 25 °C using a Bruker Avance 500 spectrometer with residual solvent peaks as references (DMSO-*d*_6_: *δ*_H_ 2.50, *δ*_C_ 39.52; CDCl_3_: *δ*_H_ 7.26, *δ*_C_ 77.16). high-resolution electrospray ionization mass spectrometry (HRESIMS) measurements were obtained on an Accurate-Mass-Q-TOF LC/MS 6520 instrument (Santa Clara, CA, USA) in positive ion mode. HPLC was performed using an Agilent 1200 Series HPLC System equipped with a diode array detector, a fraction collector, and an Agilent ZORBAX Eclipse XDB-C8 column (250 × 9.4 mm, 5 µm).

### 3.2. Microbial Material

Strain IMCAS180035 was isolated from a mud sample collected from the intertidal zones of the Yellow Sea in Qingdao, China, and grown on a potato dextrose agar plate at 28 °C. This strain was identified as *Aspergillus* sp. based on gene sequence analysis of the internal transcribed spacer (ITS) (Supplementary Figure S29) using a conventional primer pair of ITS4 (5′-TCCTCCGCTTATTGATATGC-3′) and ITS5 (5′-GGAAGTAAAAGTCGTAACAAGG-3′). The strain was deposited in the China General Microbiological Culture Collection Center (CGMCC No. 3.20170), Beijing, China, with the GenBank (NCBI) accession number MW015145.

### 3.3. Fermentation, Extraction, and Purification

The spore of *Aspergillus* sp. IMCASMF180035 stored in −80 °C was inoculated on a potato dextrose agar plate and incubated at 28 °C for 7 days; then, the fungal colony was cut into 1 cm^2^ with a sterilized knife and placed into ten 1 L conical flasks, each containing a solid medium consisting of 200 g of rice and 150 mL of distilled water. The inoculated flasks were incubated stationary at 28 °C for 30 days. The cultures and medium of *Aspergillus* sp. IMCASMF180035 were extracted three times by EtOAc:MeOH (80:20), and the combined extracts were reduced to dryness in vacuo to yield brown residue. The residue was resuspended into 500 mL of distilled water and partitioned by EtOAc. Then, the EtOAc layer was dried in vacuo to yield a dark residue (4.68 g). The EtOAc fraction was subjected to reduced pressure silica gel chromatography (50 × 70 mm column, TLC H silica) using a stepwise gradient of 80–100% hexane/CH_2_Cl_2_ and then 0–90% MeOH/CH_2_Cl_2_ to afford 15 fractions. Fraction F was purified on a Sephadex LH-20 column using an elution of CH_2_Cl_2_:MeOH (2:1) to give five subfractions, and F3 was further separated by HPLC (Agilent ZORBAX Eclipse XDB-C8, 250 × 9.4 mm, 5 μm column, 3.0 mL/min), eluting with 75% MeOH/H_2_O to yield compound **6** (3.2 mg). Fraction G was fractionated on a Sephadex LH-20 column using an elution of CH_2_Cl_2_:MeOH (2:1) to give four subfractions (G1–G4). G2 was further purified by HPLC (Agilent ZORBAX Eclipse XDB-C8, 250 × 9.4 mm, 5 μm column, 3.0 mL/min), eluting by 70% MeOH/H_2_O to yield compounds **2** (1.5 mg), **3** (1.2 mg), and **7** (1.0 mg). Fraction J was subjected to a Sephadex LH-20 column using an elution of CH_2_Cl_2_:MeOH (2:1) to give nine subfractions (J1–J9). J8 was further fractionated by HPLC (Agilent ZORBAX Eclipse XDB-C8, 250 × 9.4 mm, 5 μm column, 3.0 mL/min), eluting with 65% MeOH/H_2_O to yield compounds **4** (1.1 mg) and **8** (2.3 mg). Fraction N was subjected to a Sephadex LH-20 column using an elution of CH_2_Cl_2_:MeOH (2:1) to give ten subfractions (N1–N10). N8 was further purified by HPLC (Agilent ZORBAX Eclipse XDB-C8, 250 × 9.4 mm, 5 μm column, 3.0 mL/min), eluting by 65% MeOH/H_2_O to yield compounds **1** (2.1 mg) and **5** (5.1 mg). All the new compounds were analyzed at the same condition to check the purity and compare the retention time (Supplementary Figures S9, S16, S22 and S28).

#### 3.3.1. Aspergiloxathene A (**1**)

Aspergiloxathene A (**1**): Light yellow powder; ^1^H and ^13^C NMR data, Table 1; HRESIMS *m/z* 559.1235 [M + H]^+^ (calcd C_30_H_22_O_11_, 559.1235).

#### 3.3.2. Δ2′-1′-dehydropenicillide (**2**)

Δ^2′^-1′-dehydropenicillide (**2**): Light yellow powder; ^1^H and ^13^C NMR data, Table 1; HRESIMS *m/z* 369.1330 [M + H]^+^ (calcd for C_21_H_21_O_6_, 369.1333).

#### 3.3.3. 5-methyl-3-methoxyepicoccone (**3**)

5-methyl-3-methoxyepicoccone (**3**): Colorless powder; ^1^H and ^13^C NMR data, Table 2; HRESIMS *m/z* 241.0705 [M + H]^+^ (calcd for C_30_H_23_O_11_, C_11_H_13_O_6_, 241.0707).

#### 3.3.4. 7-carboxy-4-hydroxy-6-methoxy-5-methylphthalide (**4**)

7-carboxy-4-hydroxy-6-methoxy-5-methylphthalide (**4**): Colorless powder; ^1^H and ^13^C NMR data, Table 2; HRESIMS *m/z* 239.0554 [M + H]^+^ (calcd for C_11_H_11_O_6_, 239.0550).

### 3.4. Antibacterial Activity Assays

The microbial inhibition assays were carried out according to the Antimicrobial Susceptibility Testing Standards outlined by the Clinical and Laboratory Standards Institute document M07-A7 (CLSI) [32] by using a penal of pathogens of *S. aureus* ATCC 25923, MRSA USA300, *E. coli* ATCC 11775, *E. faecium* ATCC 19434, *P. aeruginosa* PAO1, and *H. pylori* G27. Briefly, the bacteria (*S. aureus*, MRSA, *E. coli*, *E. faecium*, and *P. aeruginosa*) were taken out from glycerol stocks and inoculated on lysogeny broth (LB) agar plate and cultured overnight at 37 °C. Then, single colonies were picked from the agar plates and adjusted to approximately 10^4^ colony-forming unit (CFU)/mL with Mueller–Hinton Broth. Two microliters of 2-fold serial dilution of each compound (in DMSO) were added to each row on a 96-well microplate containing 100 μL of bacterial suspension in each well. Vancomycin and ciprofloxacin were used as positive controls, and DMSO was used as a negative control. The 96-well plate was incubated at 37 °C aerobically for 24 h. Anti-*H. pylori* (G27) was prepared in a 96-well microtiter plate containing 100 µL of Brain Heart Infusion (BHI) broth supplemented with 10% fetal calf serum (FCS). Metronidazole was used as a positive control. The liquid culture of 2-day-old *H. pylori* was diluted 10 times in BHI broth to yield a final concentration of 5 × 10^5^ to 1 × 10^6^ CFU/mL. Two microliters of 2-fold serial dilution of each compound (in DMSO) were added into each well of the testing plates. The testing plates were incubated in a microaerophilic atmosphere at 37 °C for 3 days. The MIC was determined to be the lowest concentration, which resulted in no visible turbidity [33,34].

## 4. Conclusions

This study reported the chemical investigation on the marine-derived fungus *Aspergillus* sp. IMCASMF180035, which resulted in the isolation and characterization of eight natural products, including one dimeric anthroquinone, aspergiloxathene A (**1**); one penicillide analogue, Δ^2′^-1′-dehydropenicillide (**2**); and two new phthalide derivatives, 5-methyl-3-methoxyepicoccone (**3**) and 7-carboxy-4-hydroxy-6-methoxy-5-methylphthalide (**4**), together with four known compounds, yicathin C (**5**), 1′-dehydropenicillide (**6**), 3-methoxyepicoccone (**7**), and 4-hydroxy-6-methoxy-5-methylphthalide (**8**). To our best knowledge, compound **1** was the first natural structure with xanthene and anthracenone moieties with an unprecedented carbon skeleton with a spiro-ring system. All the isolated compounds were evaluated against a panel of bacteria, such as *S. aureus*, methicillin-resistant *S. aureus*, *E. coli*, *E. faecium*, *P. aeruginos*, and *H. pylori*. Compound **1** displayed potential antibacterial activity against *S. aureus* and MRSA, with MIC values of 5.60 and 22.40 mM, respectively. The difference in MICs between *S. aureus* and MRSA may due to the broad spectrum drug resistance of MRSA strain USA 300 to different classes of antibiotics, such as erythromycin, levofloxacin, clindamycin, tetracycline, and azithromycin [35,36]. Compounds **2** and **6** exhibited moderate antibacterial activities against *H. pylori*, with MIC values of 21.73 and 21.61 mM, respectively.

## Figures and Tables

**Table 1:** ^1^H (500 MHz) and ^13^C (125 MHz) nuclear magnetic resonance (NMR) data of **1** and **2**.

Position	1 (DMSO-*d*_6_)		Position	2 (CDCl_3_)	
	δ_C_, Type	δ_H_ (*J* in Hz)		δ_C_, Type	δ_H_ (*J* in Hz)
1	156.6, C		1	118.0, CH	6.94 (d, *J =* 8.5 Hz)
2	122.6, C		2	134.8, CH	7.72 (d, *J =* 8.5 Hz)
3	143.2, C		3	134.0, C	
4	123.5, CH	6.28 (s)	4	156.9, C	
4a	150.6, C		4a	121.6, C	
4b	153.5, C		5	166.4, C	
5	111.7, CH	5.90 (d, *J* = 2.0 Hz)	7	69.1, CH_2_	5.11 (s)
6	165.6, C		7a	125.9, C	
7	101.3, CH	6.19 (d, *J* = 2.0 Hz)	8	121.2, CH	6.40 (s)
8	163.3, C		9	135.6, C	
8a	109.7, C		10	117.8, CH	6.87 (s)
8b	114.1, C		11	147.4, C	
9	45.6, C		11a	141.0, C	
10	190.2, C		12a	154.0, C	
11	167.5, C		13	64.3, CH_3_	3.91 (s)
12	19.8, CH_3_	2.10 (s)	1’	190.7, C	
1’/8’	125.9, C		2’	124.1, CH	6.66 (s)
2’/7’	115.5, CH	6.15 (s)	3’	158.8, C	
3’/6’	144.1, C		4’	21.7, CH_3_	2.26 (s)
4’/5’	131.0, C		5’	28.3, CH_3_	2.01 (s)
4’a/4’b	137.3, C		1’’	21.0, CH_3_	2.25 (s)
8’a/8’b	117.7, C				
9’/10’	19.9, CH_3_	1.34 (s)			
3’/6’-OH		8.95 (s)			
4’/5’-OH		9.00 (s)			

**Table 2:** ^1^H (500 MHz) and ^13^C (125 MHz) NMR data of **3** and **4**.

Position	3 (DMSO-*d*_6_)		4 (DMSO-*d*_6_)	
	δ_C_, Type	δ_H_ (*J* in Hz)	δ_C_, Type	δ_H_ (*J* in Hz)
1	168.7, C		168.8, C	
3	100.5, CH	6.31 (s)	68.0, CH_2_	5.26 (s)
3a	122.5, C		119.8, C	
4	144.0, C		150.7, C	
5	141.0, C		124.8, C	
6	151.1, C		155.9, C	
7	116.1, C		118.7, C	
7a	119.0, C		128.8, C	
8	55.2, CH_3_	3.40 (s)	9.8, CH_3_	2.16 (s)
9	60.3, CH_3_	3.74 (s)	61.8, CH_3_	3.73 (s)
10	9.5, CH_3_	2.31 (s)	166.4, C	
4-OH		9.71 (s)		
6-OH		9.33 (s)		

## Data Availability

The data are contained within the text.

## Supplementary Materials

The following are available online at https://www.mdpi.com/article/10.3390/antibiotics10040377/s1, Figures S1–S28: HRESIMS, 1D and 2D NMR, and HPLC profile for compounds **1**–**4**, Figure S29: Neighbor-joining phylogenetic tree of IMCASMF180035.
