# Supplementary material for: Antibacterial Secondary Metabolites from Marine-Derived Fungus Aspergillus sp. IMCASMF180035
Source: Antibiotics (Basel). 2021 Apr 3;10(4):377. doi: 10.3390/antibiotics10040377 (PMC8066187; doi:10.3390/antibiotics10040377)
Supplement: Supplementary file 1 [file antibiotics-10-00377-s001.pdf]

SUPPLEMENTARY MATERIAL

Antibacterial Secondary Metabolites from Marine-derived Fungus *Aspergillus* sp. IMCASMF180035

Fuhang Song[1,*] Rui Lin[2], Na Yang[3,4], Jia Jia[5], Shangzhu Wei[2], Jiahui Han[2], Jiangpeng Li[2], Hongkai Bi[5] and Xiuli Xu[2,*]

Table of Contents

Figure S1. HRESIMS spectrum for **1** 3

Figure S2. ^{1}H NMR spectrum (500 MHz, DMSO-d_6) of **1** 3

Figure S3. ^{13}C NMR spectrum (125 MHz, DMSO-d_6) of **1** 4

Figure S4. HSQC spectrum (500 MHz, DMSO-d_6) of **1** 4

Figure S5. ^{1}H -^{1}H COSY spectrum (500 MHz, DMSO-d_6) of **1** 5

Figure S6. HMBC spectrum (500 MHz, DMSO-d_6) of **1** 5

Figure S7. Expansion of HMBC spectrum (500 MHz, DMSO-d_6) of **1** 6

Figure S8. Expansion of HMBC spectrum (500 MHz, DMSO-d_6) of **1** 6

Figure S9. HPLC profile of **1** 7

Figure S10. HRESIMS spectrum for **2** 7

Figure S11. ^{1}H NMR spectrum (500 MHz, $CDCl_3$) of **2** 8

Figure S12. ^{13}C NMR spectrum (125 MHz, $CDCl_3$) of **2** 8

Figure S13. HSQC spectrum (500 MHz, $CDCl_3$) of **2** 9

Figure S14. ^{1}H -^{1}H COSY spectrum (500MHz, $CDCl_3$) of **2** 9

Figure S15. HMBC spectrum (500MHz, $CDCl_3$) **2** 10

Figure S16. HPLC profile of **2** 10

Figure S17. HRESIMS spectrum for **3** 11

Figure S18. ^{1}H NMR spectrum (500 MHz, DMSO-d_6) of **3** 11

Figure S19. ^{13}C NMR spectrum (125 MHz, DMSO-d_6) of **3** 12

Figure S20. HSQC spectrum (500 MHz, DMSO-d_6) of **3** 12

Figure S21. HMBC spectrum (500 MHz, DMSO-d_6) of **3** 13

Figure S22. HPLC profile of **3** 13

Figure S23. HRESIMS spectrum for **4** 14

Figure S24. ^{1}H NMR spectrum (500 MHz, DMSO-d_6) of **4** 14

Figure S25. ^{13}C NMR spectrum (125 MHz, DMSO-d_6) of **4** 15

Figure S26. HSQC spectrum (500 MHz, DMSO-d_6) of **4** 15

Figure S27. HMBC spectrum (500 MHz, DMSO-d_6) of **4** 16

Figure S28. HPLC profile of **4** 16

Figure S29. Neighbor-joining phylogenetic tree of strain IMCASMF180035 17

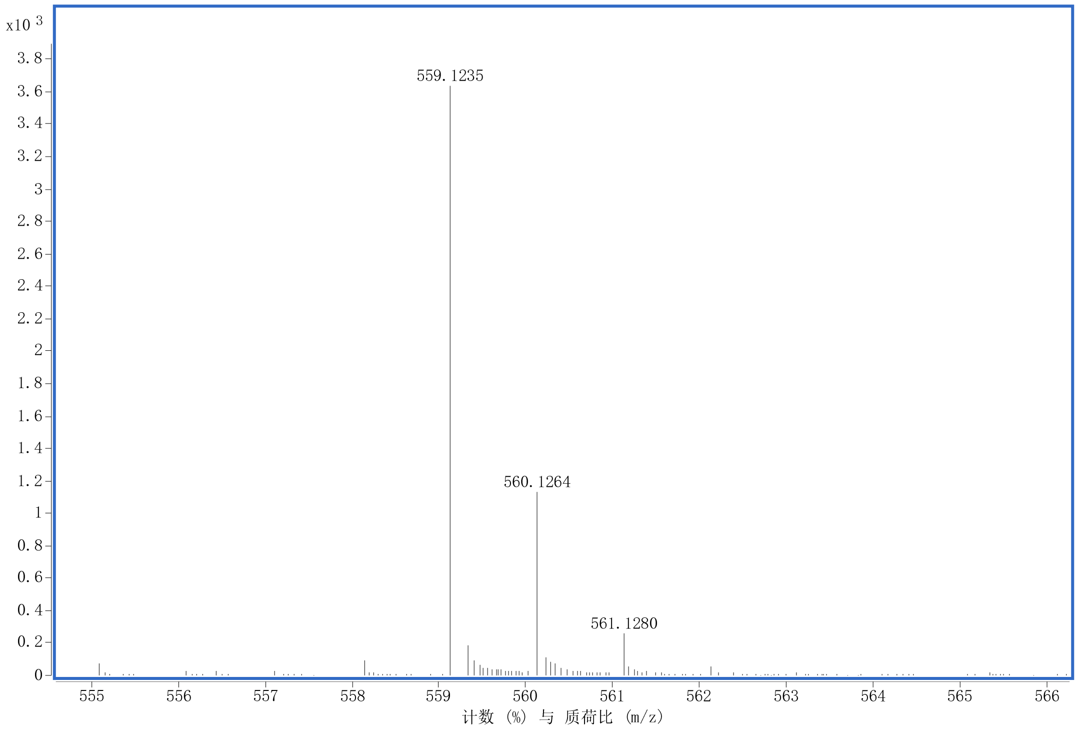

Figure S1. HRESIMS spectrum for compound **1**

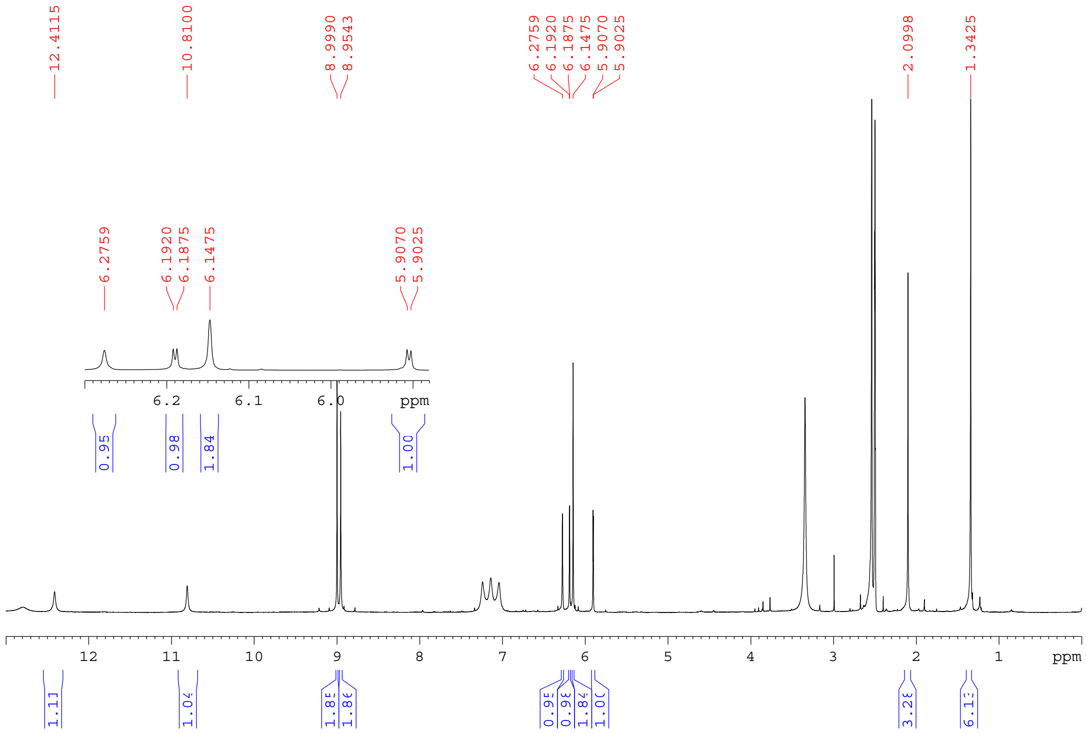

Figure S2. ^{1}H NMR spectrum (500 MHz, DMSO-d_6) of **1**

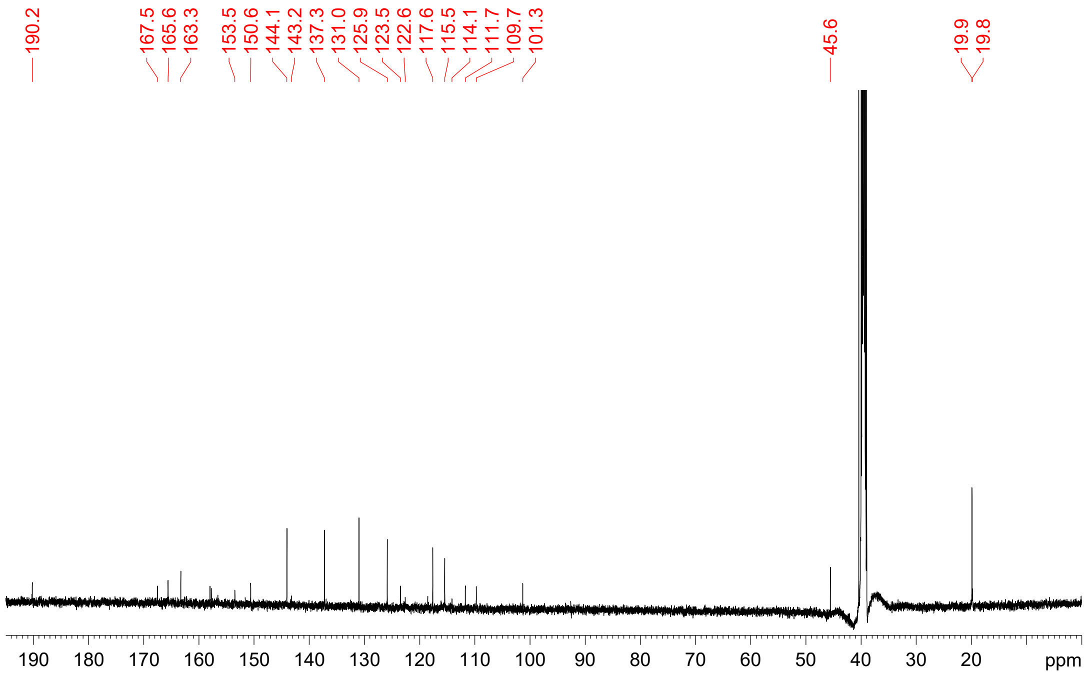

Figure S3. ^{13}C NMR spectrum (125 MHz, DMSO-d_6) of **1**

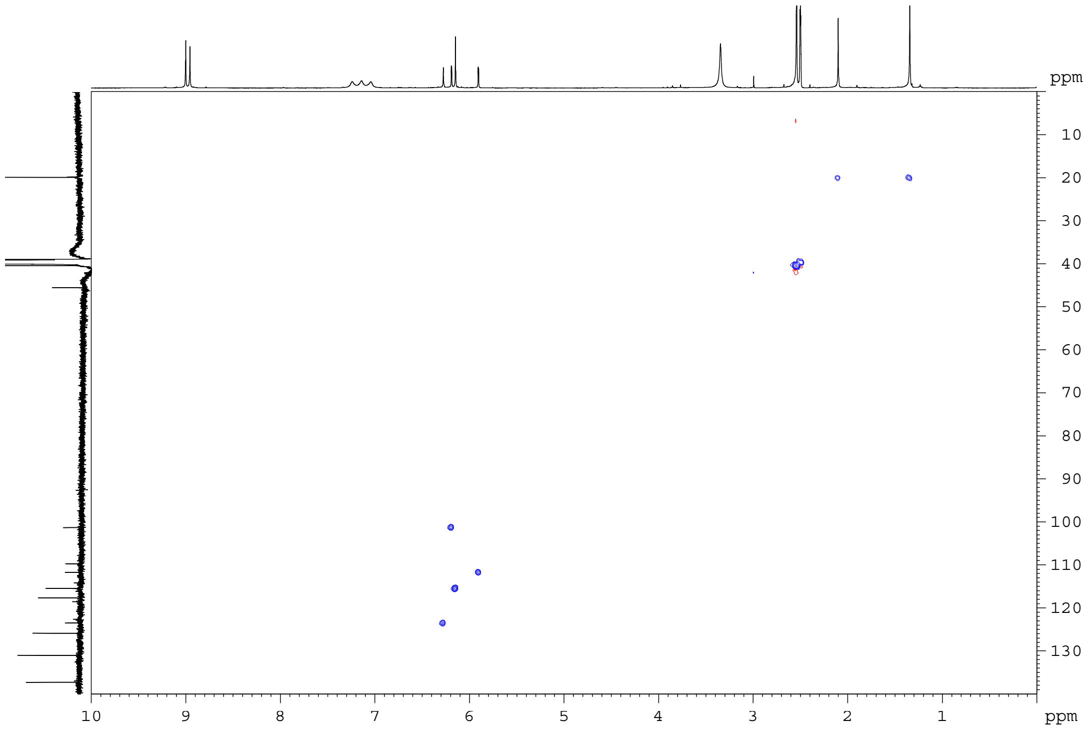

Figure S4. HSQC spectrum (500 MHz, DMSO-d_6) of **1**

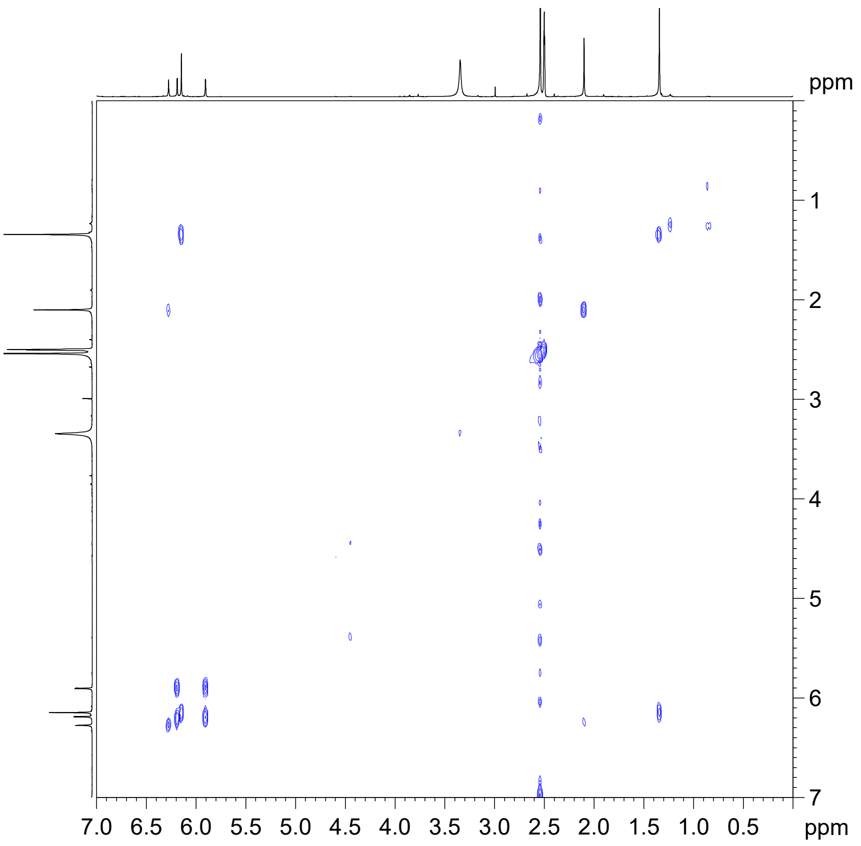

Figure S5. ^{1}H -^{1}H COSY spectrum (500 MHz, DMSO-d_6) of **1**

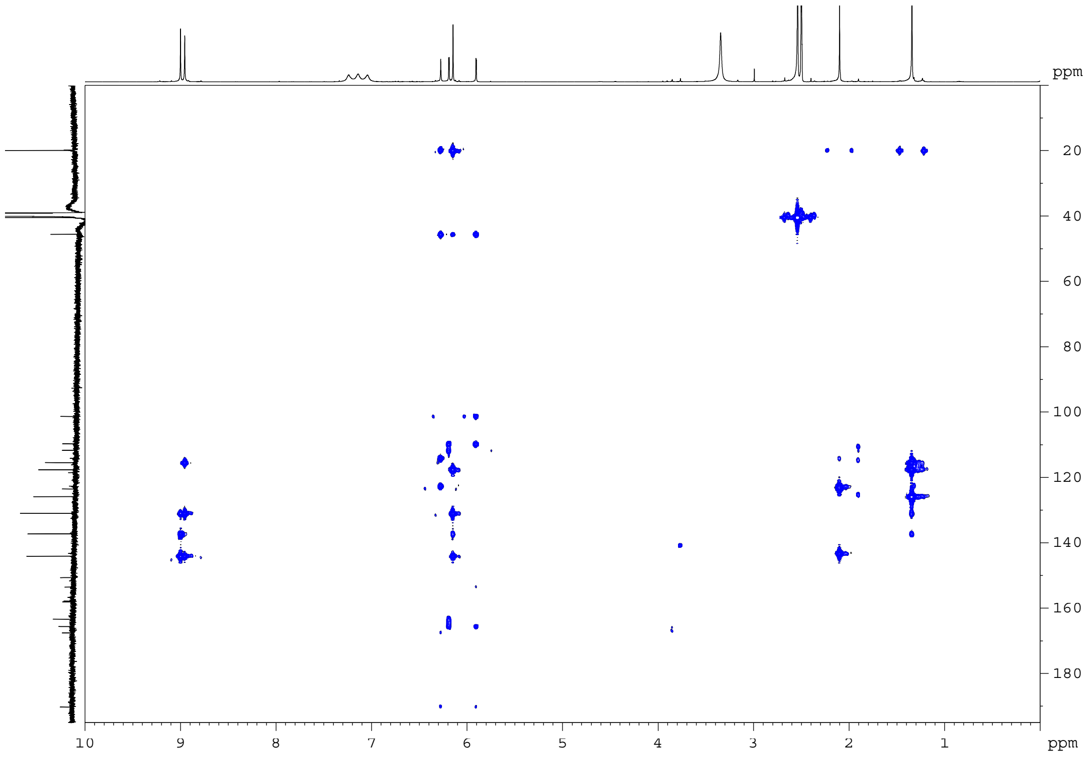

Figure S6. HMBC spectrum (500 MHz, DMSO-d_6) of **1**

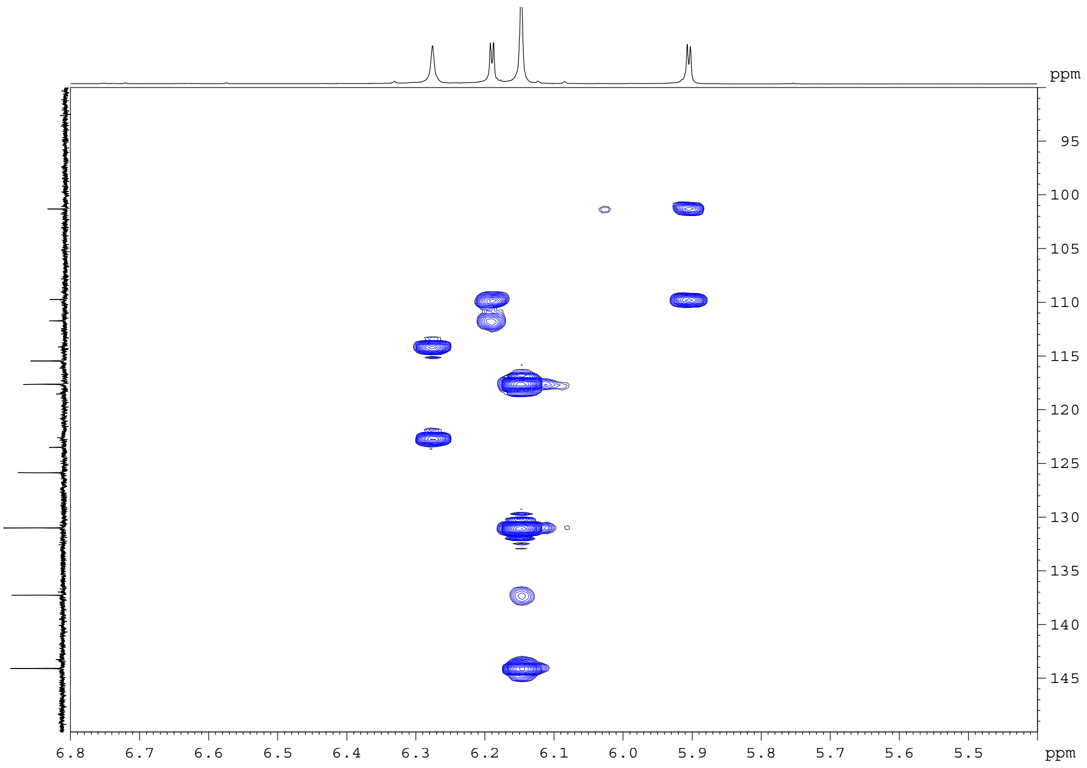

Figure S7. Expansion of HMBC spectrum (500 MHz, DMSO-d_6) of **1**

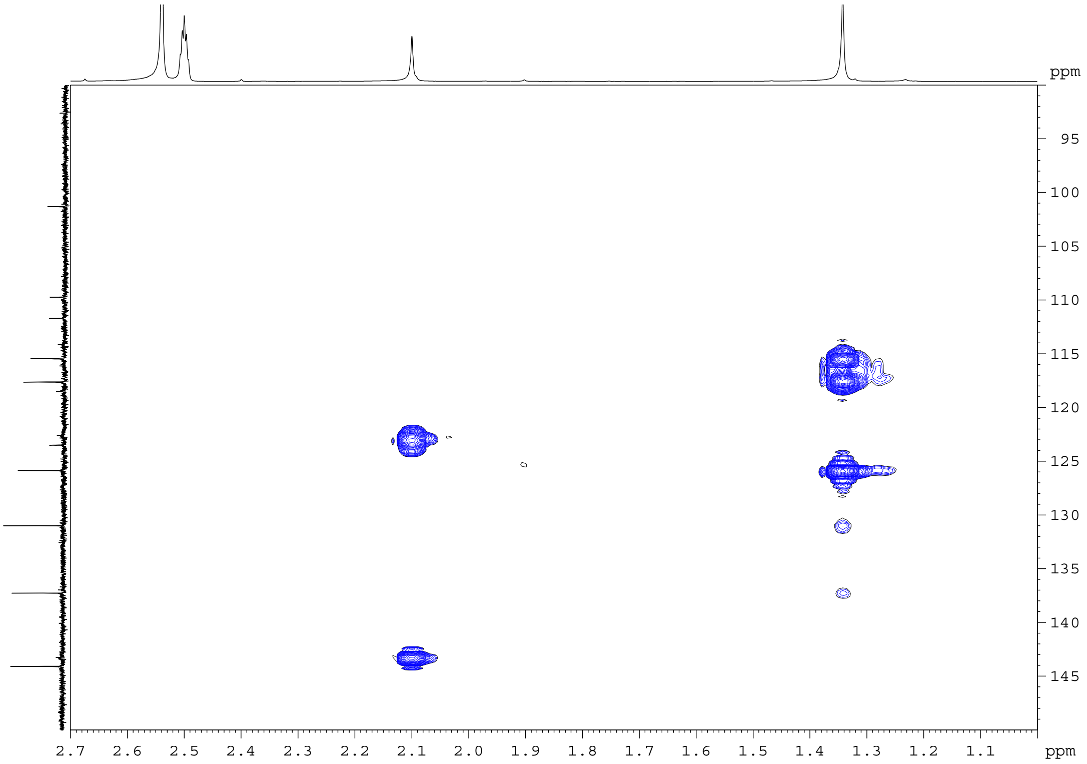

Figure S8. Expansion of HMBC spectrum (500 MHz, DMSO-d_6) of **1**

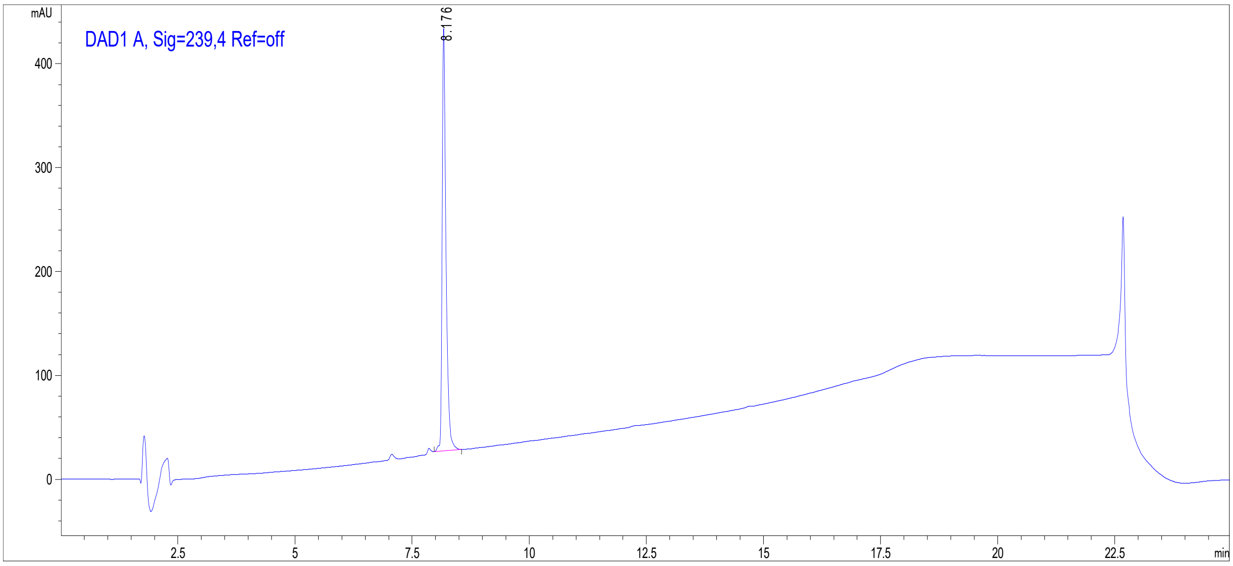

Figure S9. HPLC profile of **1**

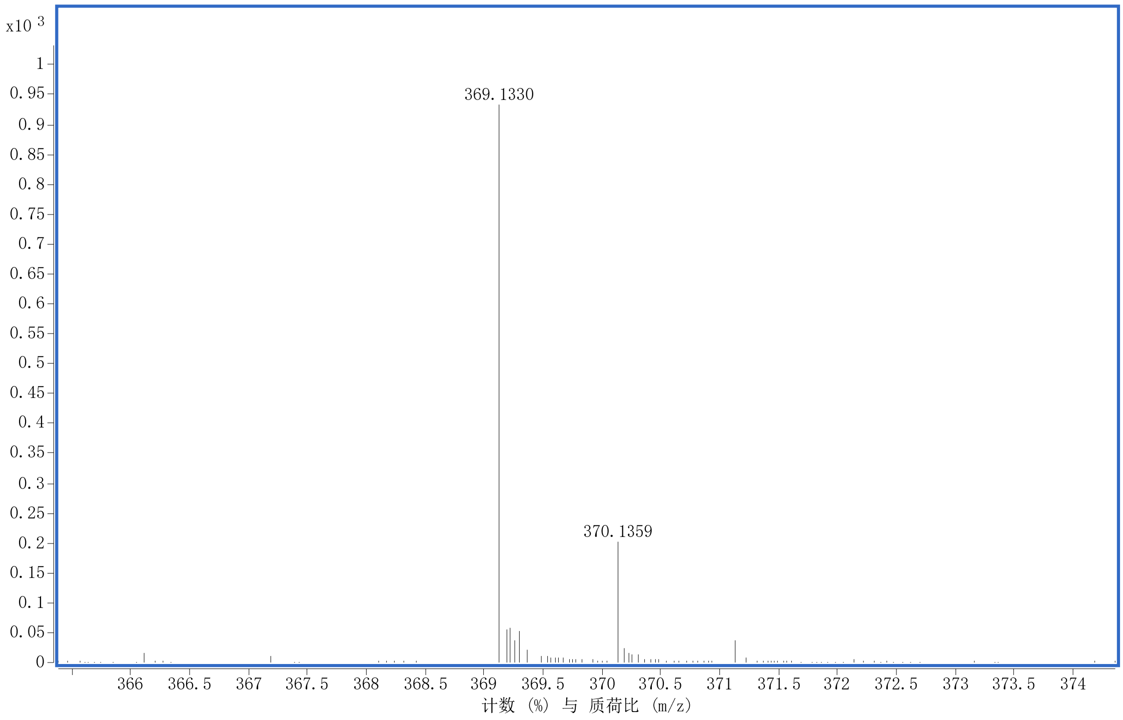

Figure S10. HRESIMS spectrum of for **2**

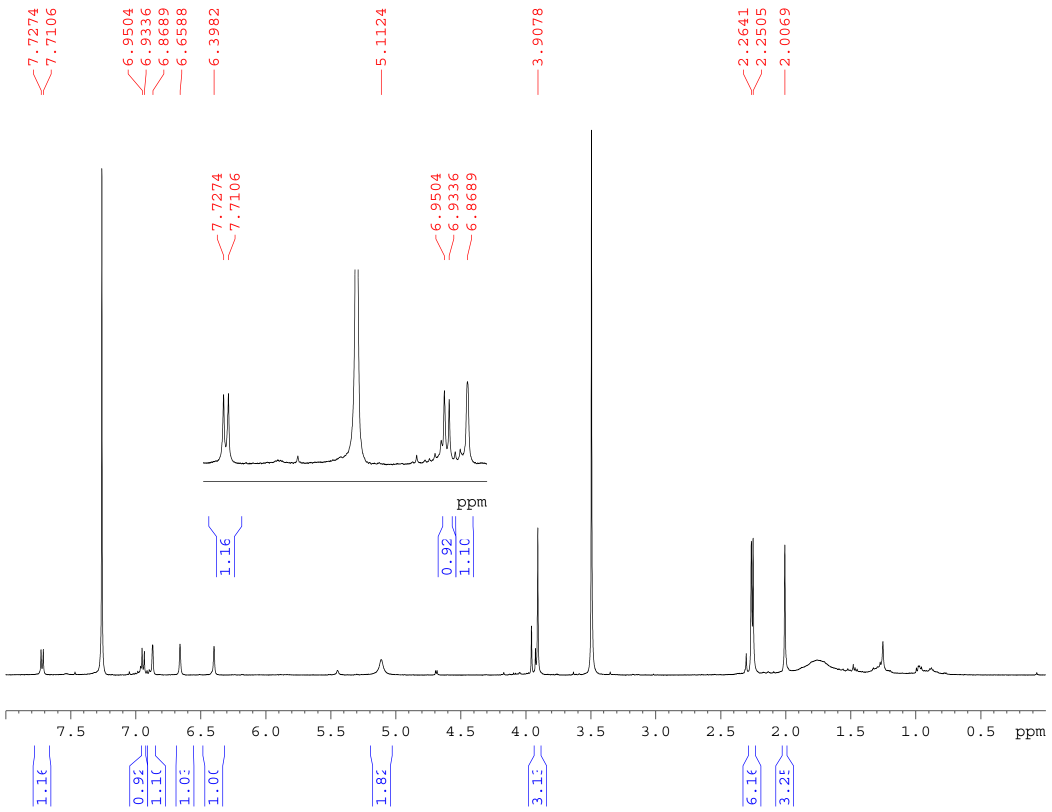

Figure S11. ^{1}H NMR spectrum (500 MHz, $CDCl_3$) of **2**

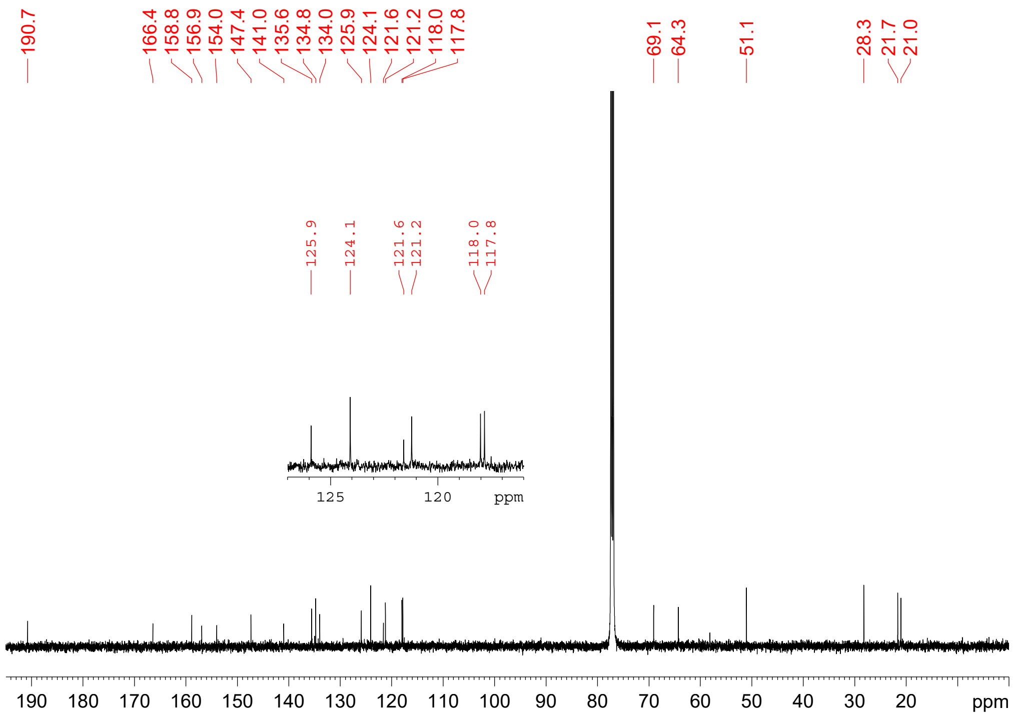

Figure S12. ^{13}C NMR spectrum (125 MHz, $CDCl_3$) of **2**

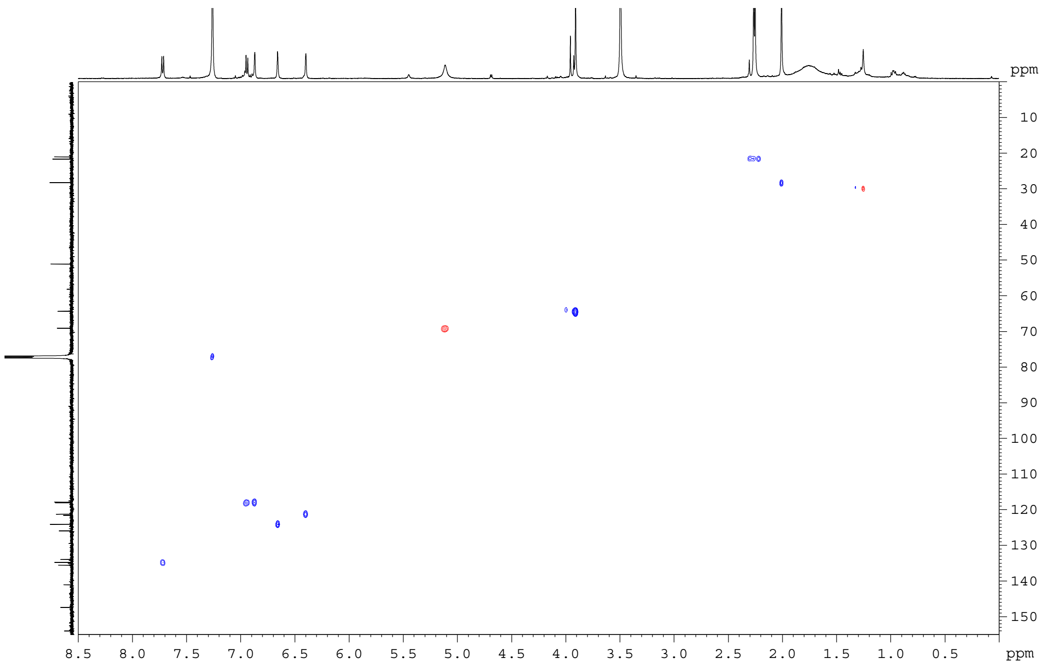

Figure S13. HSQC spectrum (500 MHz, $CDCl_3$) of **2**

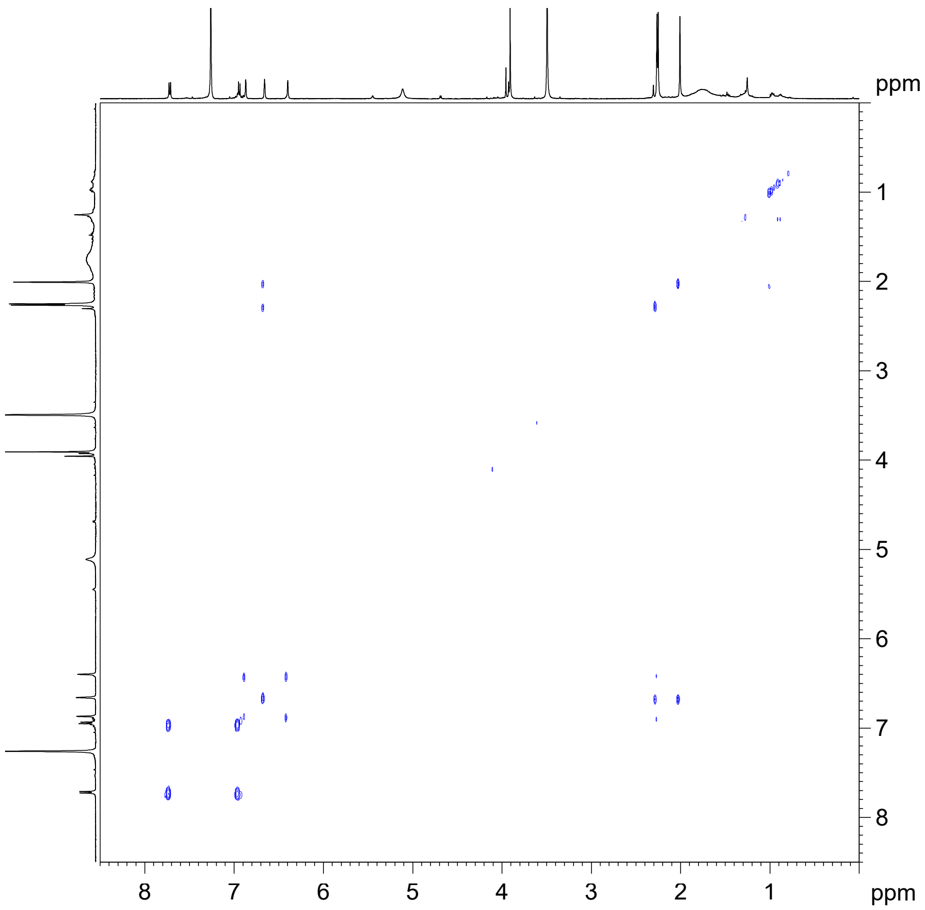

Figure S14. ^1H-^1H COSY spectrum (500MHz, $CDCl_3$) of **2**

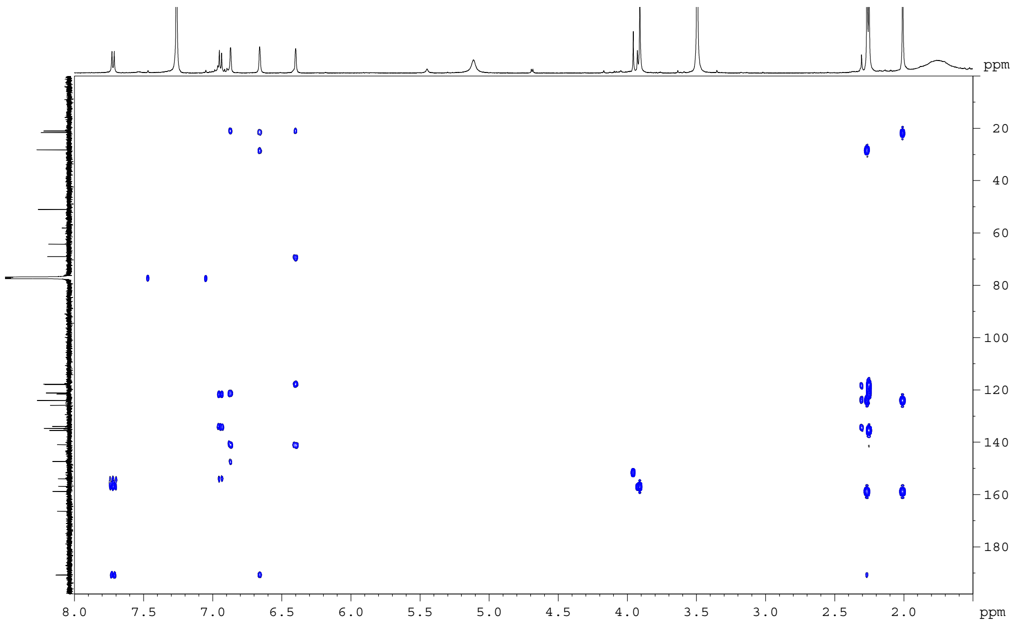

Figure S15. HMBC spectrum (500 MHz, $CDCl_3$) of **2**

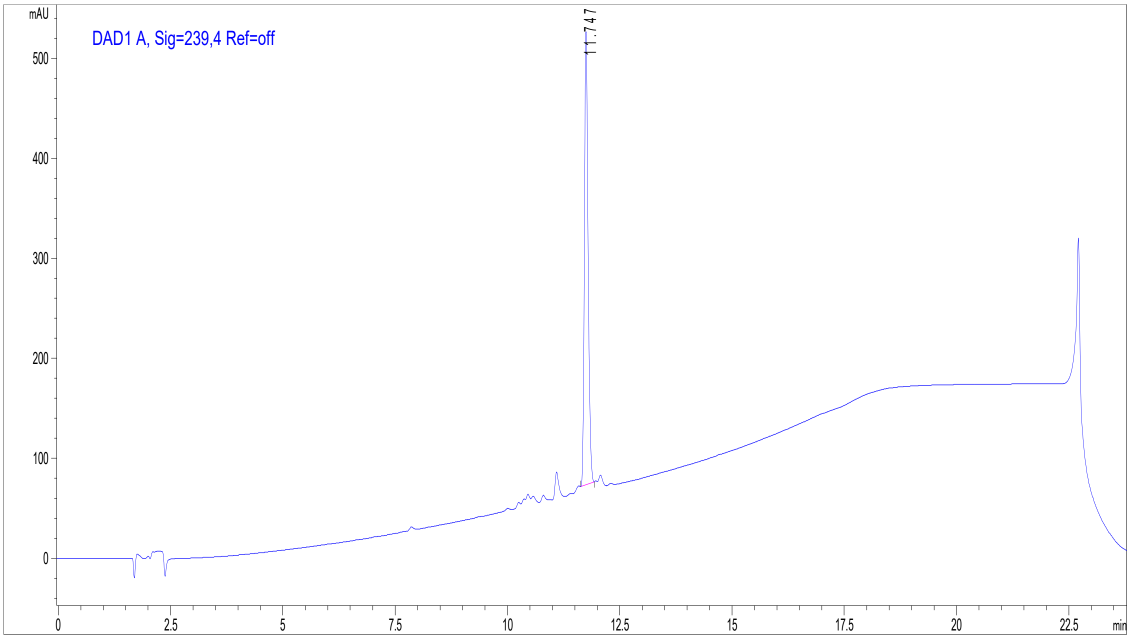

Figure S16. HPLC profile of **2**

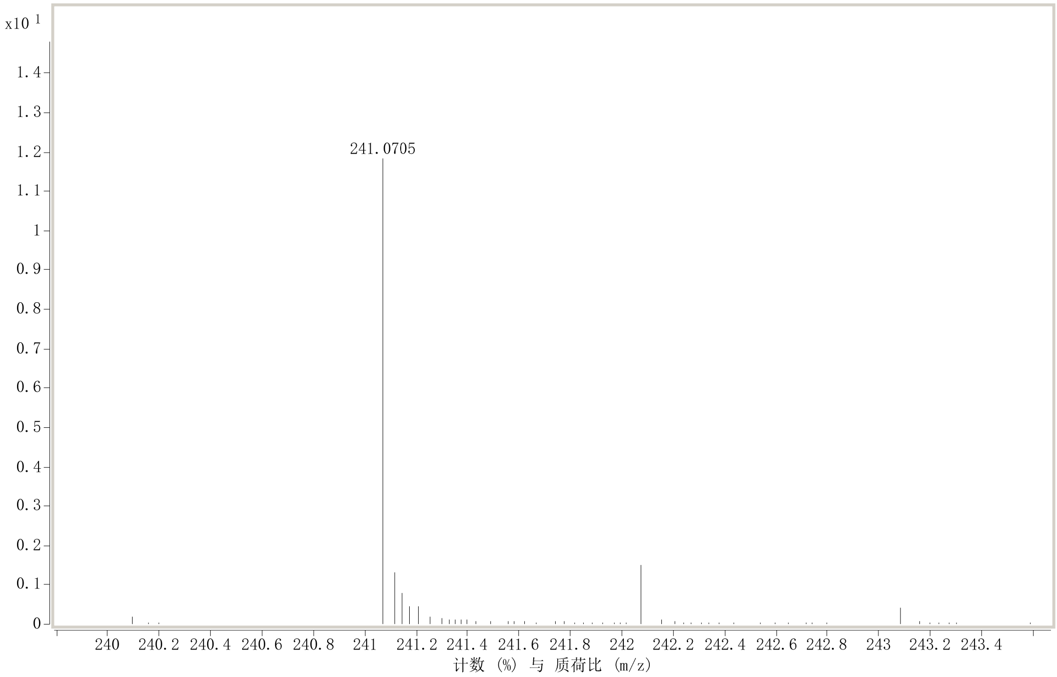

Figure S17. HRESIMS spectrum for **3**

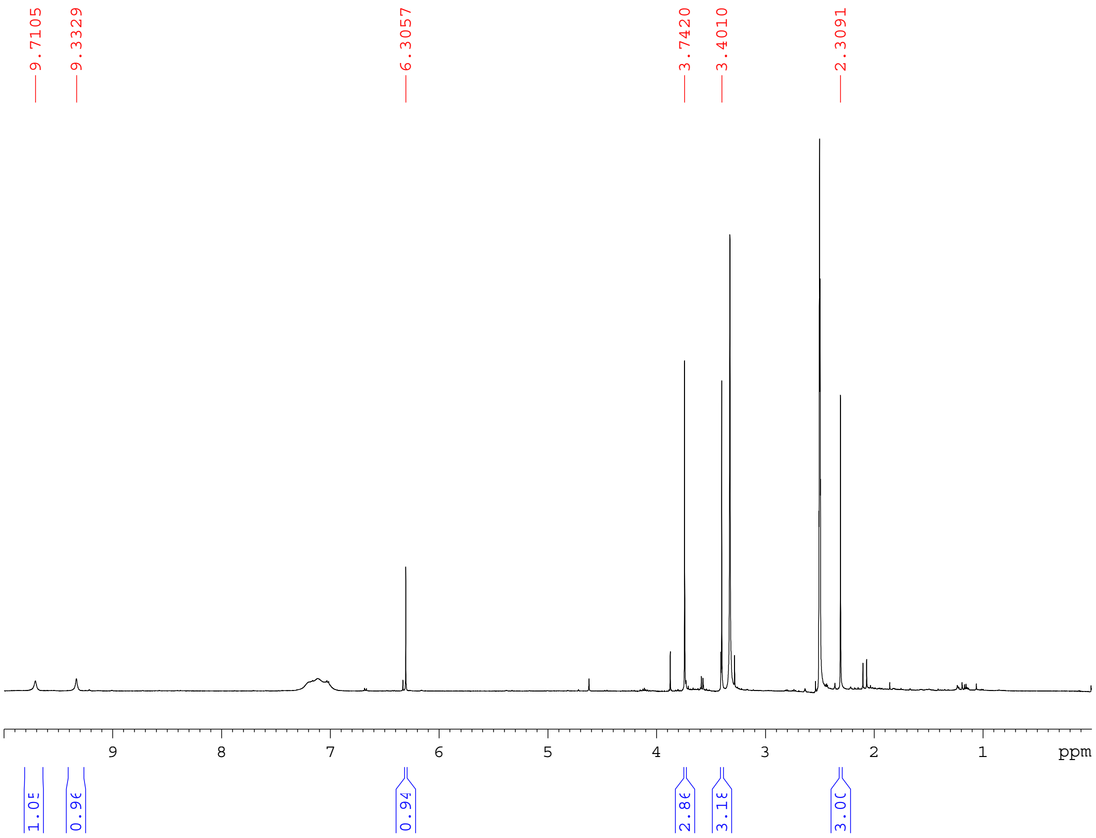

Figure S18. ^{1}H NMR spectrum (500 MHz, DMSO-d_6) of **3**

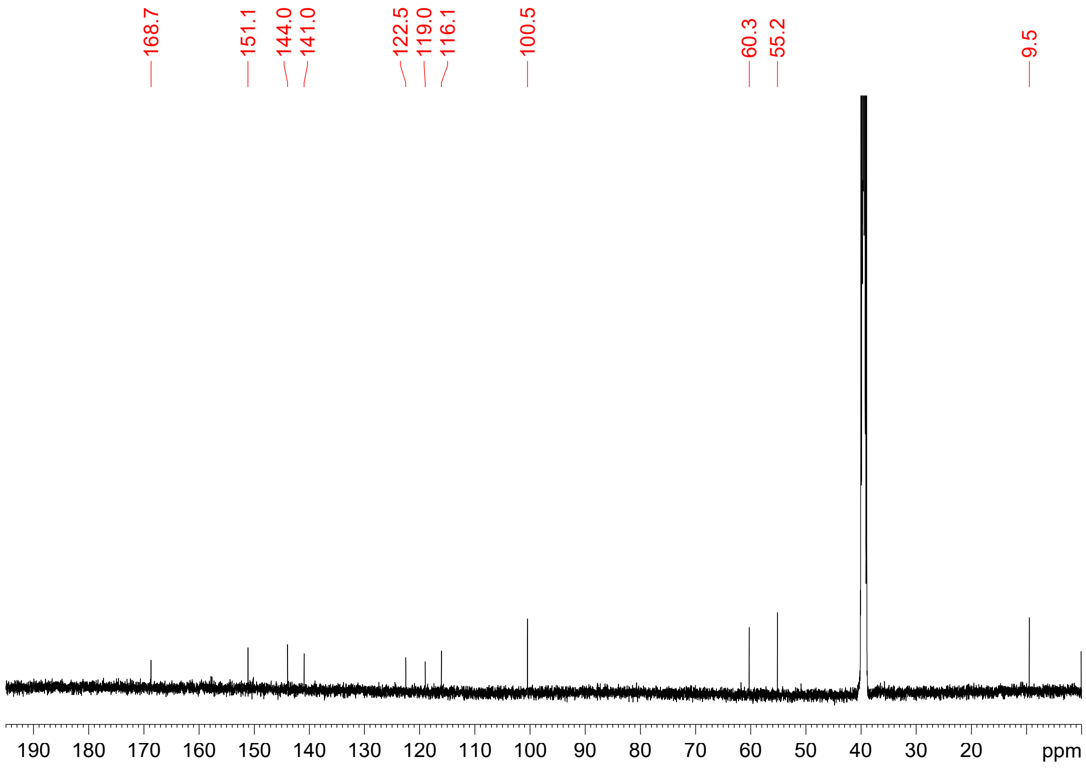

Figure S19. ^{13}C NMR spectrum (125 MHz, DMSO-d_6) of **3**

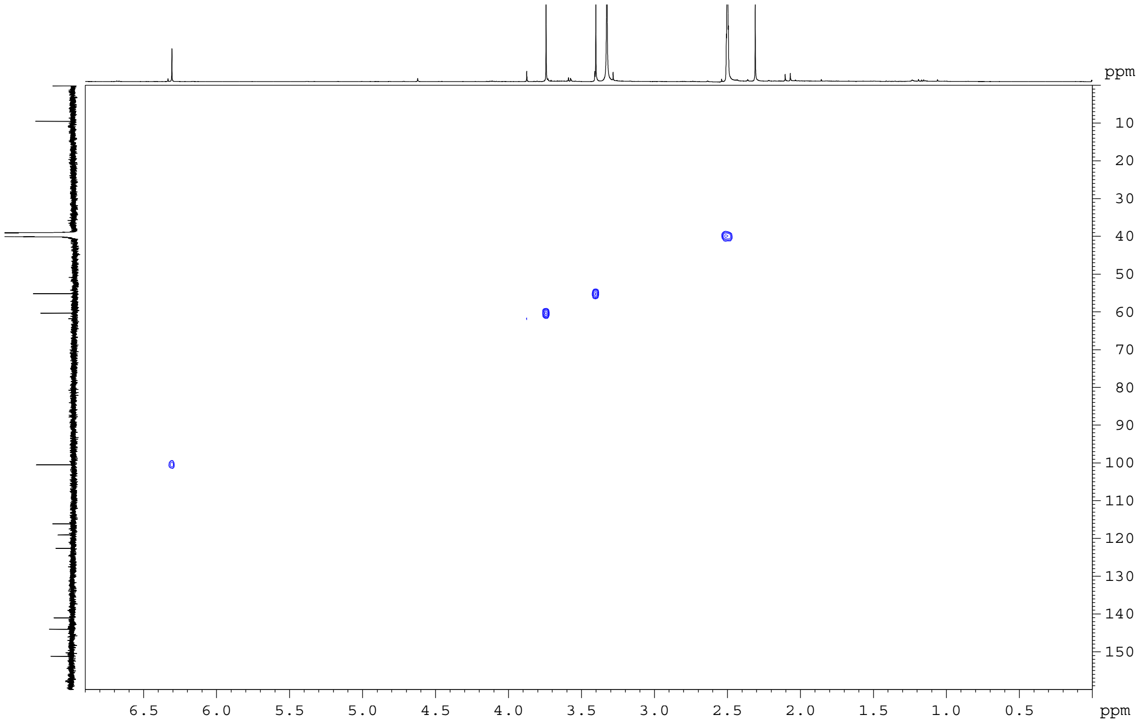

Figure S20. HSQC spectrum (500 MHz, DMSO-d_6) of **3**

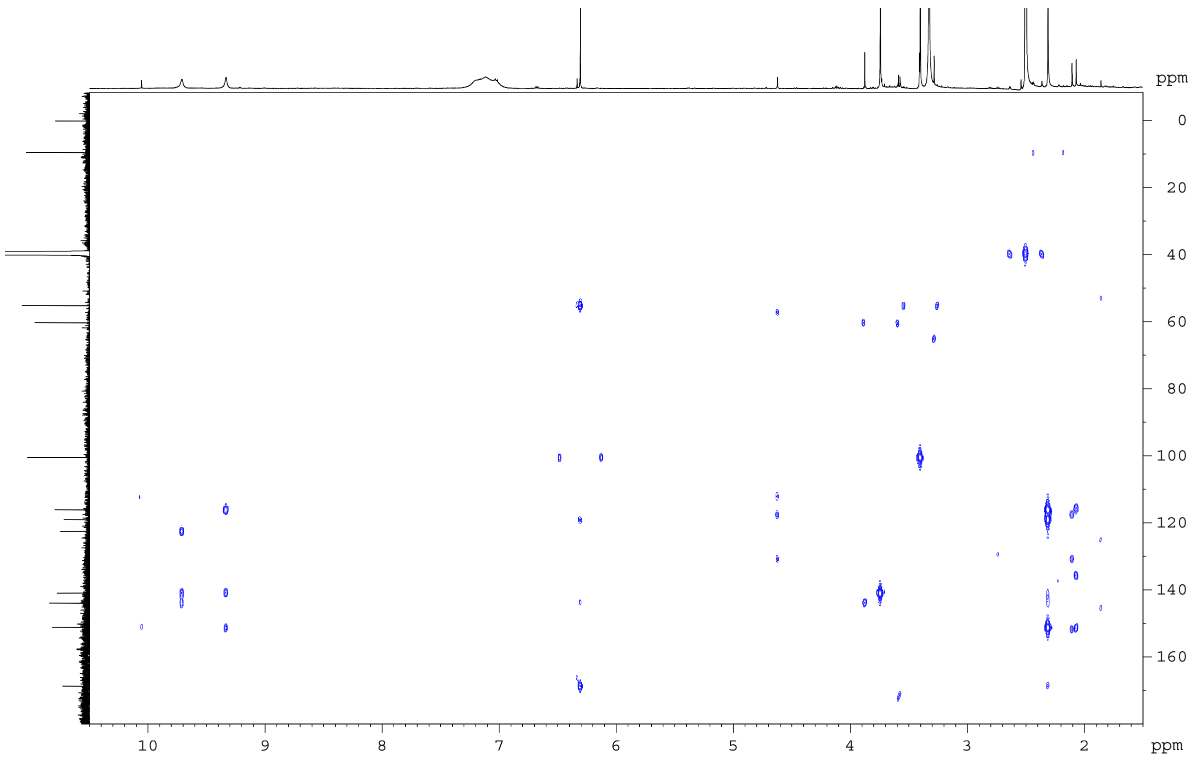

Figure S21. HMBC spectrum (500 MHz, DMSO-d_6) of **3**

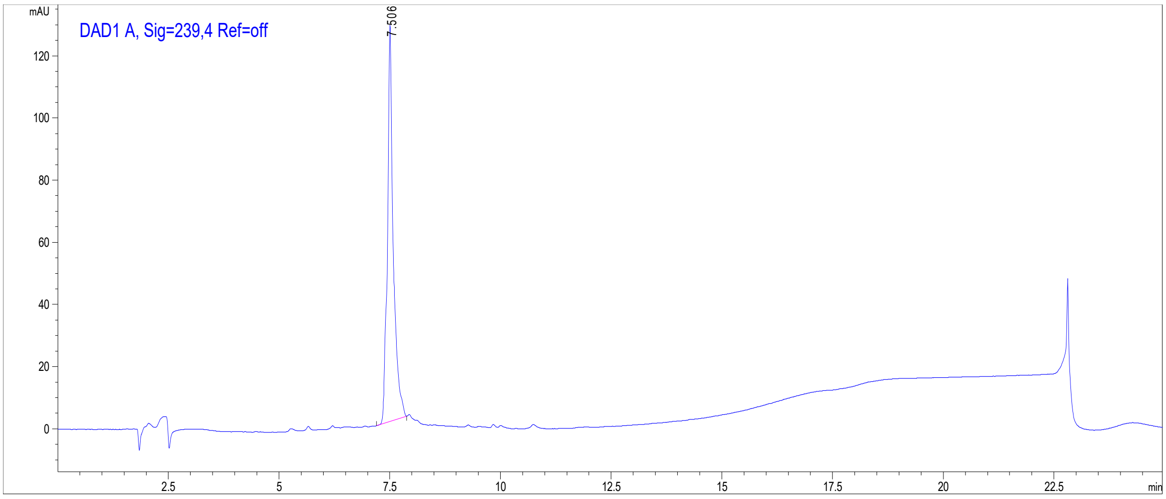

Figure S22. HPLC profile of **3**

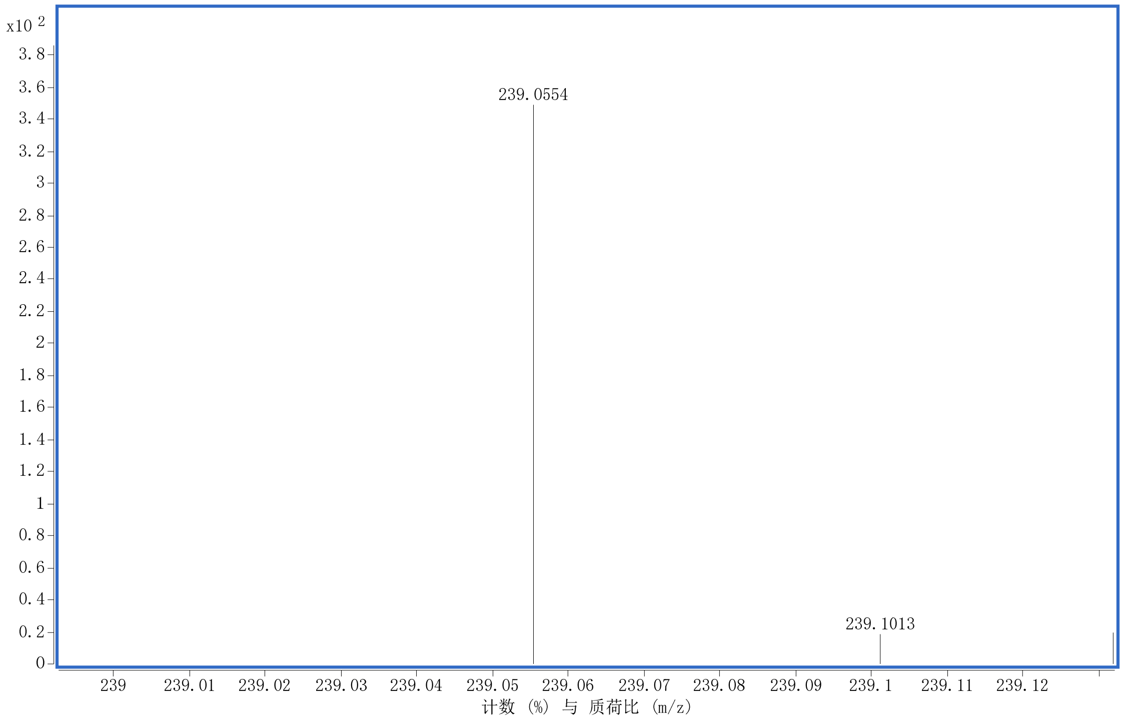

Figure S23. HRESIMS spectrum for **4**

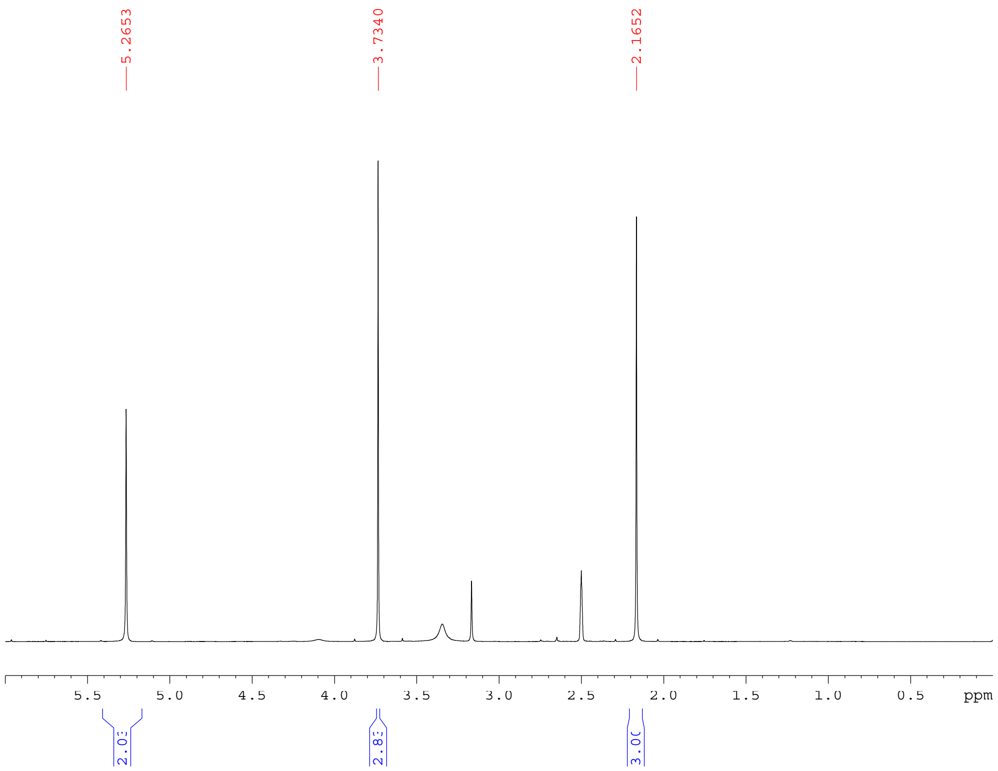

Figure S24. ^{1}H NMR spectrum (500 MHz, DMSO-d_6) of **4**

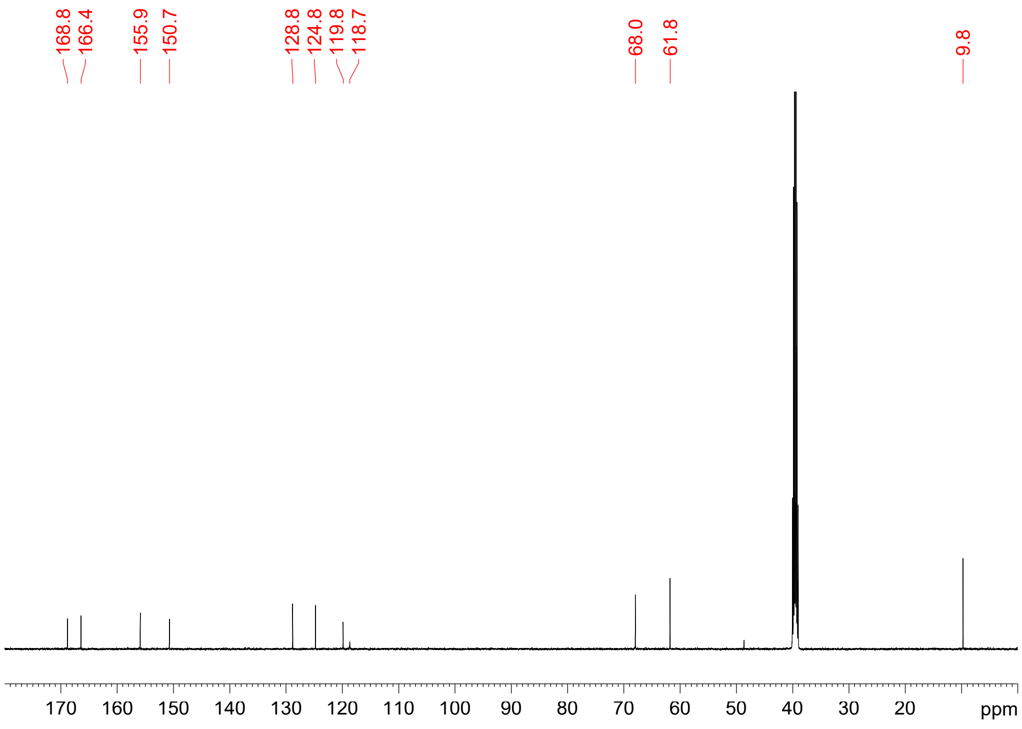

Figure S25. ^{13}C NMR spectrum (125 MHz, DMSO-d_6) of **4**

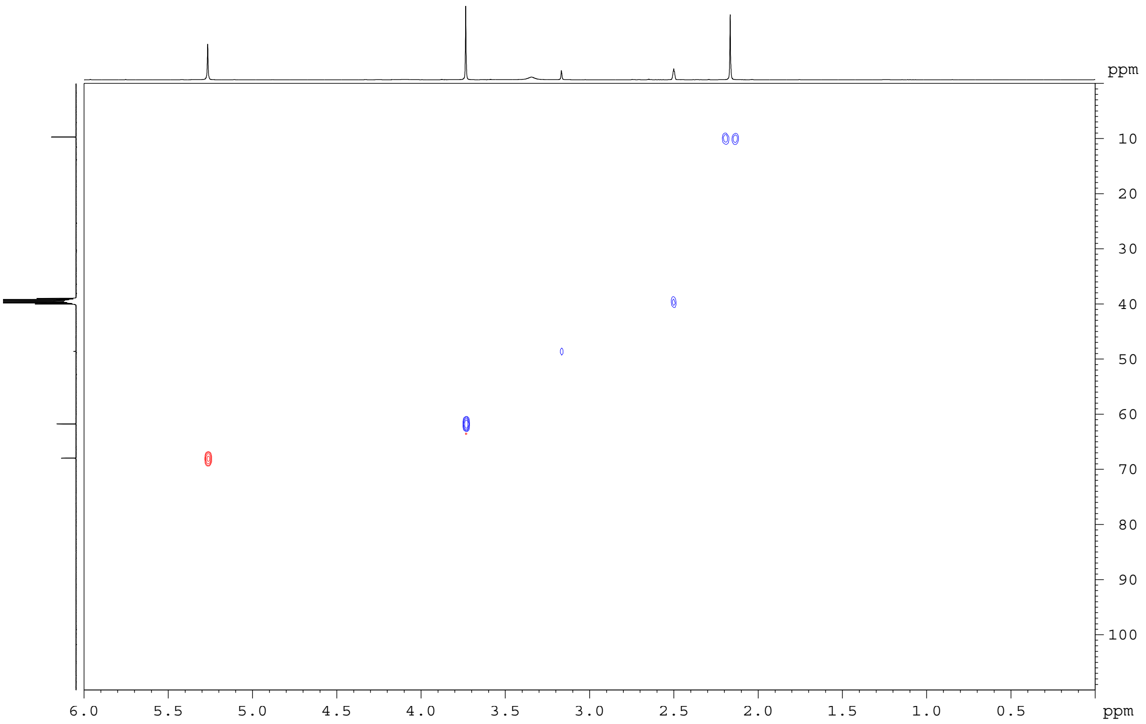

Figure S26. HSQC spectrum (500 MHz, DMSO-d_6) of **4**

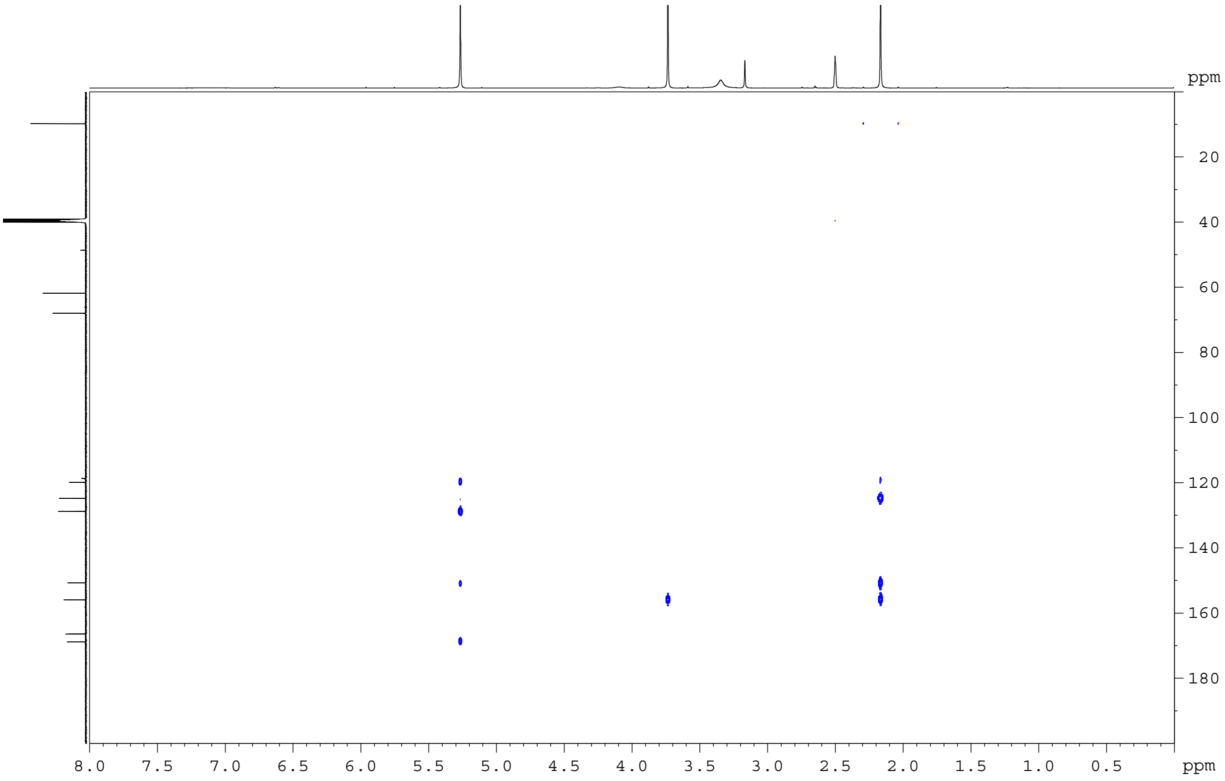

Figure S27. HMBC spectrum (500 MHz, DMSO-d_{6}3) of **4**

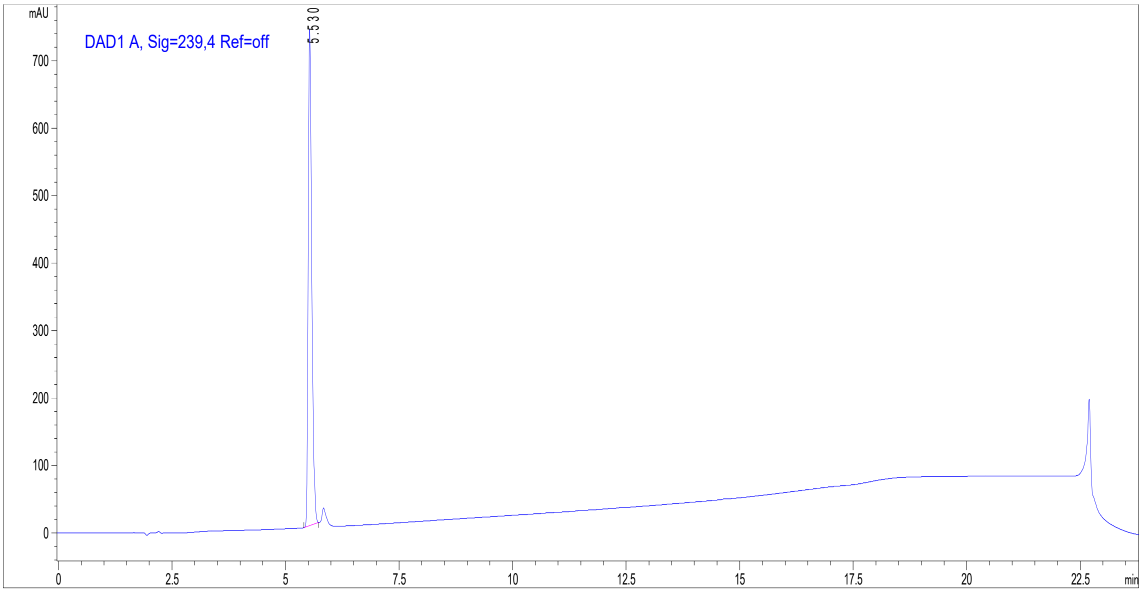

Figure S28. HPLC profile of **4**

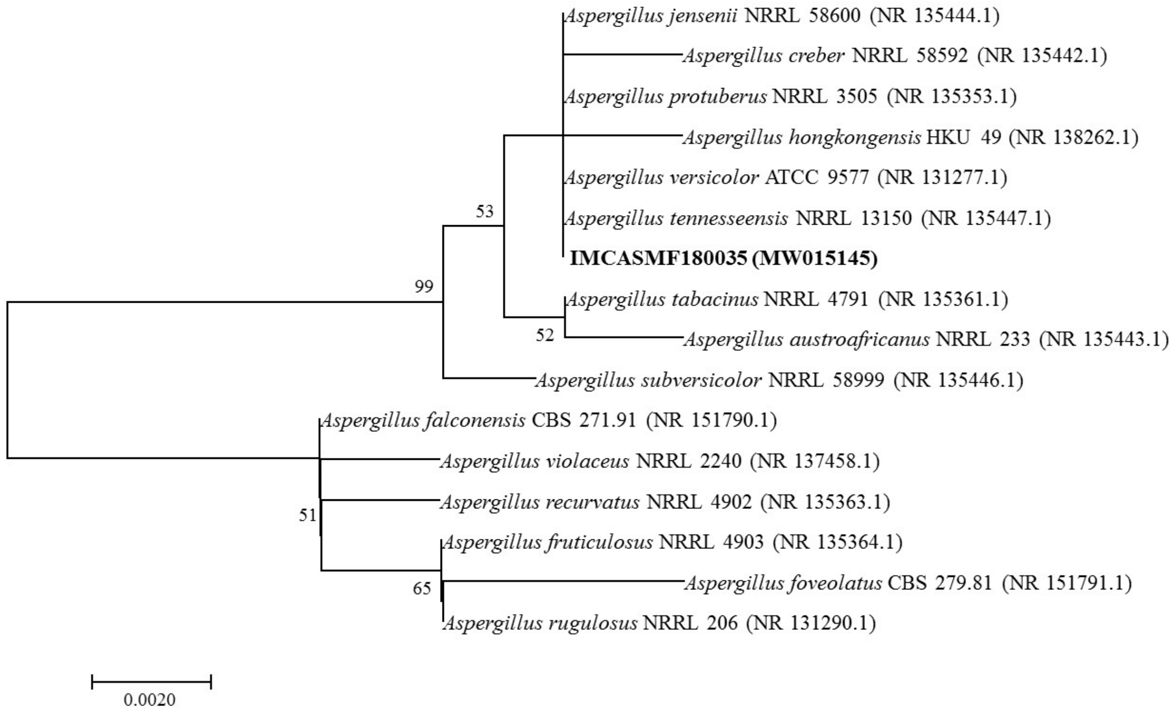

Figure S29. Neighbor-joining phylogenetic tree of IMCASMF180035 and its most related type strains based on internal transcribed spacer region (ITS) from NCBI ITS database. Numbers at nodes indicate levels of bootstrap support (%) based on a neighbor joining analysis of 1,000 resampled datasets; only values >50 % are given. NCBI accession numbers are given in parentheses. Bar 0.002 nucleotide substitutions per site.
